# Identification of the 3-amino-3-carboxypropyl (acp) transferase enzyme responsible for acp^3^U formation at position 47 in *Escherichia coli* tRNAs

**DOI:** 10.1093/nar/gkz1191

**Published:** 2019-12-21

**Authors:** Britta Meyer, Carina Immer, Steffen Kaiser, Sunny Sharma, Jun Yang, Peter Watzinger, Lena Weiß, Annika Kotter, Mark Helm, Hans-Michael Seitz, Peter Kötter, Stefanie Kellner, Karl-Dieter Entian, Jens Wöhnert

**Affiliations:** 1 Institute for Molecular Biosciences, Goethe-Universität Frankfurt, Max-von-Laue-Str. 9, 60438 Frankfurt/M., Germany; 2 Department of Chemistry, Ludwig-Maximilians-Universität München, Butenandtstr. 5, 81377 Munich, Germany; 3 Department of Cell Biology and Neurosciences, Rutgers University, Piscataway, NJ 08854, USA; 4 Institute of Pharmacy and Biochemistry, Johannes-Gutenberg-Universität Mainz, Staudingerweg 5, 55128 Mainz, Germany; 5 Institute for Geosciences, Research Unit Mineralogy, and Frankfurt Isotope and Element Research Center (FIERCE), Goethe-Universität Frankfurt, Altenhöferallee 1, 60438 Frankfurt/M., Germany; 6 Center for Biomolecular Magnetic Resonance (BMRZ), Goethe-Universität Frankfurt, Max-von-Laue-Str. 9, 60438 Frankfurt/M., Germany

## Abstract

tRNAs from all domains of life contain modified nucleotides. However, even for the experimentally most thoroughly characterized model organism *Escherichia coli* not all tRNA modification enzymes are known. In particular, no enzyme has been found yet for introducing the acp^3^U modification at position 47 in the variable loop of eight *E. coli* tRNAs. Here we identify the so far functionally uncharacterized YfiP protein as the SAM-dependent 3-amino-3-carboxypropyl transferase catalyzing this modification and thereby extend the list of known tRNA modification enzymes in *E. coli*. Similar to the Tsr3 enzymes that introduce acp modifications at U or m^1^Ψ nucleotides in rRNAs this protein contains a DTW domain suggesting that acp transfer reactions to RNA nucleotides are a general function of DTW domain containing proteins. The introduction of the acp^3^U-47 modification in *E. coli* tRNAs is promoted by the presence of the m^7^G-46 modification as well as by growth in rich medium. However, a deletion of the enzymes responsible for the modifications at position 46 and 47 in the variable loop of *E. coli* tRNAs did not lead to a clearly discernible phenotype suggesting that these two modifications play only a minor role in ensuring the proper function of tRNAs in *E. coli*.

## INTRODUCTION

In all domains of life, the biological function of RNAs is often associated with the presence of posttranscriptional chemical modifications. These modifications influence the stability, the folding pathways, the structure and the function of RNAs by e.g. modulating hydrogen bonding capabilities, stacking and hydrophobic interactions between nucleotides or the local charge distribution (1). Ribosomal RNAs (rRNAs) and transfer RNAs (tRNAs) contain particularly large numbers of modified nucleotides. Most modifications of nucleotides in rRNA are chemically simple. The most frequent nucleotide modifications in rRNAs are methylations of ribose 2′-hydroxyl groups and the isomerization of uridines to pseudouridines. Other chemically simple modifications in rRNA are methylations or acetylations of exocyclic or endocyclic nitrogen atoms in the nucleobases. The chemically most complex modification found for rRNA nucleotides so far is an *N*1-methyl-*N*3-(3-amino-3-carboxypropyl) pseudouridine (m^1^acp^3^Ψ) present at a single conserved position in the small ribosomal subunit RNA of eukaryotes (2). In archaea such as *Haloferax volcanii* an *N*3-(3-amino-3-carboxypropyl)-uridine (acp^3^U) nucleotide is found in an equivalent position (3).

In tRNAs, the frequency of posttranscriptional modifications and also their chemical variety and complexity is much higher than in rRNAs. The chemical structures of tRNA modifications, their enzymatic synthesis and their biological impact were extensively studied in the past decades. Today, >85 chemically different tRNA modifications are known, and due to novel and more sensitive analytical methods their number is still increasing (4–6). Nucleotide modifications in tRNAs are particularly prevalent in the anticodon stem–loop (ASL) (7). Most of the prokaryotic and eukaryotic tRNAs contain a modified nucleotide at position 34—the wobble position of the tRNA anticodon. Modifications at this position directly influence the decoding specificity and the stability of the codon–anticodon base pairing between mRNA and tRNA in the ribosomal A-site and thereby the fidelity of translation (8). A second ‘hotspot’ for nucleotide modifications in the ASL is position 37 directly adjacent to the anticodon. In all kingdoms of life, a wide variety of purine modifications is commonly observed at this position that modulate the structure and dynamics of the ASL and stabilize mRNA:tRNA interactions during decoding (8). A loss of modifications in this position for instance enhances the frequency of translational frame shifting (9). Nucleotide modifications in other parts of the tRNA are important for proper folding (10) or tertiary structure stabilization. Some modifications such as the dihydrouridine (D) at position 20 in the D-loop, the ribothymidine (T) at position 54 and the pseudouridine (Ψ) at position 55 in the T-loop are widely conserved among tRNAs. At many other positions, the presence and the chemical nature of modifications can be highly variable between different tRNAs and different species. Interestingly, despite decades of tRNA research a complete or nearly complete set of tRNA sequences with their respective modifications is only available for a very small number of model organisms such as the bacteria *Escherichia coli, Bacillus subtilis, Lactococcus lactis* and *Mycoplasma capricolum*, the archaeon *Haloferax volcanii* and the eukaryote *Saccharomyces cerevisiae* (11–16).

Most modifications are introduced into their target RNAs by specialized site-specific modification enzymes or enzyme cascades. An exception is the formation of pseudouridines and the methylation of 2′-hydroxyl groups in the rRNAs of archaea and eukaryotes where sequence-specific guide RNAs cooperate with an unspecific pseudouridine synthase or methyl transferase complex to introduce these modifications (17,18). Thus, all organisms must encode a significant number of different RNA modification and in particular tRNA modification enzymes. For model organisms such as *E. coli* and *S. cerevisiae*, it has been shown that up to 10% of their genomic coding capacity is devoted to tRNA modification enzymes (19) again illustrating the biological importance of RNA modifications. However, the genes encoding tRNA modification enzymes are often not essential. Nevertheless the lack of certain modification enzymes causes diverse phenotypes in translation initiation, accuracy and efficiency, especially under stress conditions (19). Furthermore, mutations leading to defects in tRNA modification enzymes of higher eukaryotes were recently linked to metabolic defects, mitochondrial phenotypes, neurodevelopmental disorders and cancer (20,21).

**Figure 1.**
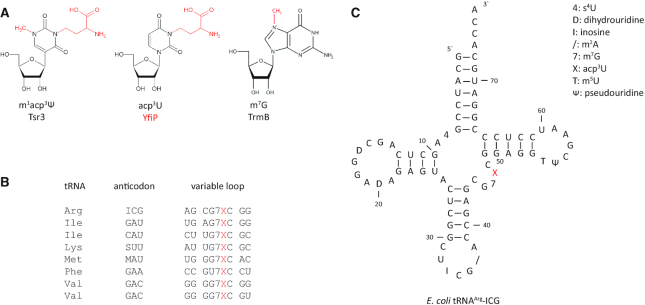
RNA acp modifications. (**A**) Modified nucleosides m^1^acp^3^Ψ (eukaryotic 18S rRNA), acp^3^U (bacterial and eukaryotic tRNA and archaeal 16S rRNA) and m^7^G together with the responsible modification enzymes discussed in this work. (**B**) Variable loop sequences of all acp^3^U containing tRNAs from *E. coli*. (**C**) Secondary structure of *E. coli* tRNA^Arg^-ICG with its posttranscriptional modifications. The acp^3^U (X) at position 47 in the variable loop is highlighted in red.

Efforts to identify tRNA modification enzymes and to link them functionally to the biosynthesis of specific tRNA modifications usually require the combination of biochemical and genetic approaches as well as comparative genomics. The precise prediction of enzyme function based on sequence data alone is normally not possible since for instance homologous proteins from the same protein family might be responsible for modifying different target sites in different organisms (19). This is exemplified e.g. by the RlmD family of methyltransferases. Here, the archaeal enzymes modify tRNAs whereas in *E. coli* the RlmD enzyme modifies 23S rRNA (22). In other cases, members of evolutionary unrelated enzyme families modify equivalent tRNA positions in different organisms due to convergent evolution. A prominent example for this situation is the widespread methylation of U54 in tRNAs at the C5 carbon yielding ribothymidine (T) at this position. The responsible enzyme is a *S*-adenosyl methionine (SAM) dependent methyltransferase belonging to the TrmA family in many Gram-negative bacteria (23). In, contrast in Gram-positive bacteria such as *B. subtilis* the same modification is introduced by an FAD containing methyltransferase of the TrmFO family which uses *N*^5^,*N*^10^-methylenetetrahydrofolate as the methyl group donor (24). It is also possible that parallel metabolic pathways contribute simultaneously to the introduction of a specific modification in the same organism (25). Thus, even for such extensively studied model organisms such as *E. coli* or *S. cerevisiae* there is not yet a complete description of all tRNA modifications and their corresponding modification enzymes. However, with regard to the so far identified tRNA modifications for *E. coli* only the enzyme(s) responsible for the introduction of the acp^3^U modification has (have) been remained unidentified. The acp^3^U nucleotide, also abbreviated as X (Figure 1A) was first isolated from *E. coli* tRNA^Phe^ and chemically characterized by Ohashi *et al.* (26) and later found in seven additional tRNA species (Figure 1B, Supplementary Figure S1, Table S1). Early on SAM was identified as an essential cofactor for introducing the acp^3^U modification as the donor of the acp group (27). However, the responsible modification enzyme was not identified.

In the eight *E. coli* tRNAs that carry an acp^3^U modification the modified nucleotide is always located at position 47 in the variable loop region in the sequence context 5′-m^7^G-acp^3^U-C-3′ (Figure 1B and C, Supplementary Figure S1). Chemical derivatization of acp^3^U with acylating agents had no severe impact on the aminoacylation or translation capability of tRNA^Phe^, indicating an at most subtle influence of this modification on the tertiary structure of the tRNA (28).

The acp^3^U modification is also present in selected eukaryotic tRNAs. However, here the modified nucleotide is not located in the variable loop but occurs instead e. g. at position 20a of the D-loop (29,30). Interestingly, the levels of acp^3^U were shown to be increased in supernatants from breast carcinoma cells, and might therefore serve as a tumor marker *in vivo* (31).

Up to now, only two evolutionary unrelated enzymes capable of transferring the acp group from SAM to RNA nucleotides have been functionally and structurally characterized. The Tyw2/Trm12 protein catalyzes an intermediate step during the synthesis of the tRNA wybutosine modification in archaea and eukaryotes. The structure of Tyw2/Trm12 is similar to Rossmann-fold RNA-methyltransferases (32). Recently, we showed that the enzyme Tsr3 catalyzes a SAM-dependent acp transfer during the hypermodification of the yeast 18S rRNA nucleotide 1191 to m^1^acp^3^Ψ. Surprisingly, structural analysis of archaeal Tsr3 homologs revealed a fold typical for SPOUT-class RNA-methyltransferases. However, a SAM binding mode of Tsr3 distinct from canonical SPOUT-class methyltransferases positions the acp group of SAM in the active site poised for reaction, whereas the methyl group of the cofactor is buried in a deep hydrophobic pocket of Tsr3 (33). The acp transfer catalyzed by Tsr3 is the final step in the biogenesis of m^1^acp^3^Ψ from uridine. Here, U1191 in yeast 18S rRNA is initially converted to pseudouridine by an H/ACA snoRNP containing the snoRNA guide snR35 (34). Subsequently, Ψ1191 is methylated at the N1 nitrogen to 1-methyl-pseudouridine (m^1^Ψ) by the SAM-dependent SPOUT-class methyltransferase Nep1 (35–37)—one of only two known pseudouridine N1 methylating enzymes (38). Interestingly, in a yeast *Δsnr35* deletion strain where U1191 is not converted to Ψ Tsr3 accepts uridine instead of the usual m^1^Ψ as the target nucleotide resulting in an acp^3^U modification of the 18S rRNA (37). Thus, while eukaryotic Tsr3 normally transfers an acp group to m^1^Ψ it also has an intrinsic ability to work as an U N3 acp transferase enzyme. Furthermore, we identified archaeal Tsr3 homologs in species such as *H. volcanii* where no genes encoding Nep1 homologs are present and where the 16S rRNA is known to contain acp^3^U instead of m^1^acp^3^Ψ (3). Structural comparisons between eukaryotic and archaeal Tsr3 proteins and functional dissection of the yeast enzyme demonstrated that the archaeal Tsr3 homologs correspond to the functional core domain of the enzyme (33). Already before the function of Tsr3 as an rRNA modification enzyme and acp transferase became clear Burroughs and Aravind (39) showed by bioinformatic analysis that Tsr3 proteins are members of a widespread protein superfamily. This so-called TDD superfamily was named after proteins of the Tsr3, DTWD1 and DTWD2 families, which all share a DTW domain due to the presence of a central D/E…X…T/S…W motif. Our structural analysis of archaeal Tsr3 homologs revealed that this motif is central to the function of Tsr3 as an acp transferase. The first amino acid of the motif (D/E) is most likely the catalytic base of the enzyme and the last amino acid of the motif (W) is responsible for creating the unique SAM-binding mode of Tsr3. Taken together; sequence comparisons and the structural analysis suggest that acp transferase activity might be common in the TDD superfamily. Interestingly, the TDD family does not only contain eukaryotic and archaeal Tsr3 homologs involved in the biosynthesis of the conserved m^1^acp^3^Ψ/acp^3^U in the small ribosomal subunit rRNA but also bacterial proteins. One of the bacterial proteins was the functionally uncharacterized *E. coli* protein YfiP. However, *E. coli* does contain neither m^1^acp^3^Ψ nor acp^3^U in its ribosomal RNAs. Instead in *E. coli* and in other bacterial 16S rRNAs the nucleotide corresponding to the eukaryotic U1191 is replaced by a methylated G (40). The only acp modified nucleotides in *E. coli* are the acp^3^U nucleotides in the variable loops of eight *E. coli* tRNAs as described above. This suggested to us that the *E. coli* YfiP protein might be a suitable candidate for the unknown U47 N3 acp transferase in *E. coli*.

In this paper, we demonstrate by a combination of genetic, biochemical and mass spectrometric approaches that the *E. coli* YfiP protein is indeed the missing tRNA acp transferase for U47 and consequently rename this protein as **t**RNA **U**47 **a**cp transferase **A** or TuaA. Furthermore, we show that the degree of the acp^3^U modification at U47 is dependent on the presence of the m^7^G modification at the preceding nucleotide G46 and in addition depends on medium conditions. However, the physiological importance of the acp^3^U modification at U47 remains unclear since a deletion strain did not show a discernible phenotype under a variety of growth and stress conditions. Overall, our identification of YfiP as the SAM-dependent acp transferase responsible for the biogenesis of acp^3^U at position 47 in *E. coli* tRNA together with our previous characterization of the DTW-domain containing Tsr3 as an m^1^Ψ acp transferase enforces the notion that acp transfer to RNA nucleotides might be a general function of DTW proteins.

## MATERIALS AND METHODS

### Growth conditions and media

*Escherichia coli* strains were grown in LB-Lennox (5 g/l NaCl) or M9 medium as indicated at 37°C. M9-glucose medium was prepared as described earlier (41). Antibiotics were used in the following concentrations: 100 μg/ml ampicillin, 25 μg/ml kanamycin. Yeast strain CEN.PK2 was grown at 30°C in YPD medium (1% yeast extract, 2% peptone, 2% glucose) or in synthetic dropout medium (0.5% ammonium sulfate, 0.17% yeast nitrogen base, 2% glucose) lacking histidine or leucine, respectively. Liquid cultures in tubes or flasks were continuously shaken at 125 rpm.

### Construction of deletion strains

All deletion strains generated in this study are listed in Supplementary Table S2. *Escherichia coli* strain BW25993 (42) was obtained from the *E. coli* Genetic Stock Center (CGSC, cgsc2.biology.yale.edu) and was used as parental strain for the construction of all deletion strains. The single and double deletion strains were constructed by the recombination method of Datsenko and Wanner (42) using the lambda red system expressed from pKD46. Deletion cassettes were amplified by PCR using pKD13 (FRT-kan^R^-FRT) as template and transformed by electroporation in BW25993. The kanamycin resistance of the deletion strains was eliminated by Flp recombinase mediated excision after transformation with pCP20. All deletion strains were verified by PCR using for the FRT-kanR-FRT deletion strains both internal and external primers to either the kanamycin resistance gene or the WT gene. Deletion strains with eliminated resistance cassette were verified with external primers only. Primers used for the construction of the deletion strains and for verification are listed in Supplementary Table S3.

### Plasmid construction and site-directed mutagenesis

Plasmids in the present study (listed in Supplementary Table S4) were constructed by an *in vivo* recombination strategy (gap-repair) in budding yeast using strain CEN.PK2 (43) with the oligonucleotides listed in Supplementary Table S3.

Specific point mutations were introduced by polymerase chain reaction (PCR) site-directed mutagenesis using the Single-Primer Reactions in Parallel (SPRINP) method and high-fidelity Phusion-DNA polymerase (NEB) (44). All plasmids carrying point mutations were sequenced, transformed in the *ΔyfiP* strain (Ec.PK6-10) and tested for complementation by primer extension. *E. coli* codon optimized genes of YfiP orthologues from *Variovorax paradoxus* (*Vp*YfiP) and *Clostridium saccharoperbutylacetonicum* (*Cs*YfiP) were synthesized by Eurofins Genomics. The synthetic genes were amplified by PCR and cloned in pPK894 (Supplementary Table S2). For overexpression and purification, the *E. coli yfiP* gene was cloned into a pET11a derivative (pPK565) with an additional sequence encoding a C-terminal 6xHis-tag resulting in plasmid pPK984.

### Isolation of tRNA

*Escherichia coli* cells were grown up to an OD_600_ of 1.0 and total RNA was isolated with phenol/ chloroform (45). 600 μg of total RNA were mixed with an equal volume of 2× TEN buffer and layered onto a 5–25% sucrose gradient in TEN buffer (10 mM Tris–HCl pH 7.8, 10 mM NaCl, 1 mM EDTA). Gradients were centrifuged at 25 000 rpm (22 h at 4°C) in a SW40 rotor using an L-70 Beckman ultracentrifuge and afterwards fractionated with an ISCO density gradient system monitoring absorbance at 254 nm. Low density fractions containing tRNAs and other small RNAs were pooled, and the RNA was precipitated with EtOH/LiCl.

### HPLC analysis of tRNA nucleosides

15–120 μg of tRNA were digested and hydrolyzed as described by Gehrke and Kuo (46). Nucleosides were separated on a Supelcosil LC-18S column (Sigma; 250 × 4.6 mm, 5 μm) with a pre-column (4.6 × 20 mm) using an ammonium phosphate/methanol gradient (37,47). For ESI-MS analysis, acp^3^U was further purified over an Eclipse XDB-C18 column (Agilent; 150 × 4.6 mm, 5 μm) with 250 mM ammonium acetate pH 6.0 as eluent A and 40% acetonitrile as eluent B (flow rate: 0.5 min/ml; up to 50% B over 10 min). After buffer evaporation and washing with 650 μl RNase-free water, samples were vacuum dried.

### Primer extension

Oligonucleotides were labeled with γ-[^32^P]-ATP (33), hybridized to 125 ng of tRNA, and primer extension was carried out as described by Sharma *et al.* (48). Samples were separated on a 6% or 8% denaturing acrylamide gel (Model S2, Biometra) in 1× TBE buffer at 80 W. After electrophoresis, the gel was transferred to 3MM paper, and signals were visualized by autoradiography. In parallel tRNAs were sequenced from template plasmids using the dideoxy cycle sequencing method (49), and the reaction products were separated together with the primer extension samples.

Alternatively, Cy5-fluorescent oligonucleotides (Eurofins) were applied for primer extension analysis following the protocol described above and for sequencing using the Thermo Sequenase Cycle Sequencing Kit (Thermo Fisher Scientific).

### YfiP overexpression and purification

*Escherichia coli* YfiP with a C-terminal hexahistidine tag (pPK984) was expressed in *E. coli* BL21(DE3) Gold (Agilent Technologies/Stratagene) in LB-medium supplemented with ampicillin (100 μg/ml). Protein expression was induced at OD_600_ ∼0.8 with 1 mM IPTG at 37°C. After 3 h, cells were harvested by centrifugation at 5000 × g and 4°C.

*Escherichia coli* cells were lysed by sonication in a buffer containing 50 mM sodium phosphate, pH 7.5, 500 mM NaCl, 10 mM imidazole, 5 mM β-mercaptoethanol, 0.1 % Triton X-100, DNAse (Roche) and Complete^®^ protease inhibitor (Roche). After sonication the lysate was cleared by centrifugation (8000 × g, 4°C, 45 min) and passed over a HisTrap HP column (GE Healthcare) in NiNTA buffer (sodium phosphate, pH 7.5, 300 mM NaCl, 2 mM β-mercaptoethanol). The recombinant protein was eluted with a linear gradient ranging from 0 to 500 mM imidazole over a volume of 100 ml. Fractions containing protein were diluted to 100 mM NaCl with Tris–HCl, pH 7.5 and further purified by cation exchange chromatography using a HiPrep 16/10 SP column (GE Healthcare) and a salt gradient from 100 mM to 1 M NaCl.

In a final step, the protein was purified on a HiPrep 16/60 Sephacryl S-100 high resolution column (GE Healthcare) equilibrated with 50 mM Tris–HCl, pH 7.5, 200 mM NaCl, 2 mM β-mercaptoethanol. All purification steps were performed at 4°C with an ÄKTA purifier (GE Healthcare).

### Isothermal titration calorimetry (ITC)

ITC measurements were performed at 20°C in 50 mM sodium phosphate, pH 7.5, 200 mM NaCl, 2 mM β-mercaptoethanol with an iTC200 calorimeter (Malvern Panalytical). 300 μl purified YfiP with a concentration of 86 μM was titrated with *S*-adenosyl methionine (SAM, 2 mM or 1.3 mM), 5′-deoxy-5′-methyl thioadenosine (MTA, 3.1 mM) or *S*-adenosyl homocysteine (SAH, 1.8 mM) by 20 injections with a 2 μl injection volume. The stirring speed of the injection syringe was set to 750 rpm. Binding constants were determined with Origin7 (Origin Lab) using a one-site binding model. The reported *K*_D_s are the averages of at least three titration experiments for each ligand.

### ICP-MS for the detection of zinc ions

Trace elements (Zn isotopes) were analysed with a single collector sector field ICP-MS system (Element XR, ThermoFisher Scientific) housed at the Frankfurt Isotope and Element Research Center (FIERCE), Goethe-Universität Frankfurt, Institute for Geosciences. Weighted samples were digested in 1 ml of 6 M HNO_3_ at 120°C for 24 h in teflon polyfluoroalkoxy (PFA) screw-top vials. Afterwards samples were dried down, dissolved and diluted with 5% HNO_3_. Measurements were performed using a glass-spray chamber fitted with an ESI microconcentric nebulizer with an uptake rate of 50 μl/min. Hot plasma conditions were used in order to minimize recombination and subsequent formation of possible molecular interferences, such as oxides. The following elements were analyzed: Cr, Mn, Fe, Co, Ni, Cu and Zn. Instrumental drift was corrected by bracketing samples with a multi-element standard. Blank correction was done by subtracting an analytical blank (chemistry blank minus background signal on doubly distilled 2% HNO_3_) and by monitoring the elemental composition of the protein buffer solution (50 mM TrisHCl, pH 7.5, 200 mM NaCl). As additional controls, the zinc content was measured for the Nob1 protein from *Pyrococcus horikoshii*, which was previously shown to contain one bound zinc ion per monomer (50), and for the protein Lpp1663 from *Legionella pneumophila*, which was shown to be zinc-free (51).

### *In vitro* activity assay

tRNA was isolated from *E. coli ΔyfiP* via a sucrose gradient as described above. 2.1 μg tRNA were incubated with purified YfiP protein (20 μM) and SAM (1 mM) in a 50 mM Tris buffer pH 7.5 including 200 mM NaCl and 100 μM ZnCl_2_ for 60 min at room temperature. 5 μl from 25 μl total reaction volume were applied to primer extension for detection of the acp^3^U-47 modification in tRNA^Arg^-ICG.

### tRNA isoacceptor purification for mass spectrometry

The procedure was adapted from Hauenschild *et al.* (52). For tRNA isoacceptor purification, pre-purified total tRNA was used. The sequences of the biotinylated 2′-deoxyoligonucleotide probes are listed in Supplementary Table S3.

### tRNA digestion for mass spectrometry

Total tRNA (∼100 ng) or purified tRNA isoacceptors were digested to single nucleosides by using 0.2 U alkaline phosphatase (Sigma-Aldrich), 0.02 U phosphodiesterase I (VWR, Radnor, Pennsylvania, USA), and 0.2 U benzonase (Sigma-Aldrich) in Tris (pH 8, 5 mM, (Sigma-Aldrich)) and MgCl_2_ (1 mM, (Sigma-Aldrich)) containing buffer. Furthermore, 0.5 μg tetrahydrouridine (Merck, Darmstadt, Germany), 1 μM butylated hydroxytoluene (Sigma-Aldrich), and 0.1 μg pentostatin (Sigma-Aldrich) were added to avoid deamination and oxidation of the nucleosides. The mixture was incubated for 2 h at 37°C and then filtered through 96-well filter plates (AcroPrep Advance 350 10 K Omega, PALL Corporation, New York, USA) at 3000 × g and 4°C for 30 min. 1/10 volume of SILIS (stable isotope labeled internal standard) as prepared in Borland *et al.* (53) was added to each filtrate before analysis by QQQ mass spectrometry.

### QQQ mass spectrometry

For quantification, a 1290 Infinity II UHPLC equipped with a DAD (Agilent Technologies, Waldbronn, Germany) was combined with an G6470A Triple Quad system and electro-spray ionization (ESI-MS, Agilent Jetstream). Optimized operating parameters were set as follows: positive ion mode, skimmer voltage 15 V, cell accelerator voltage 5 V, N_2_ gas temperature 230°C and N_2_ gas flow 6 l/min, sheath gas (N_2_) temperature 400°C with a flow of 12 l/min, capillary voltage of 2500 V, nozzle voltage of 0 V and the nebulizer at 40 psi. The instrument was operated in dynamic MRM mode and the individual mass spectrometric parameters for the nucleosides are given in Supplementary Table S7. Mobile phase A was 5 mM NH_4_OAc (≥99%, HiPerSolv CHROMANORM^®^, VWR), brought to pH 5.3 with glacial acetic acid (≥99%, HiPerSolv CHROMANORM^®^, VWR). Mobile phase B was pure acetonitrile (Roth, LC–MS grade, purity $ \ge$ 99.95). A Synergi Fusion-RP column (Phenomenex^®^, Torrance, California, USA; Synergi^®^ 2.5 μm Fusion-RP 100Å, 150 × 2.0 mm) at 35°C and a flow rate of 0.35 ml/min was used. Gradient elution started with 100% A for 1 min, increased to 10% B after 5 min, and to 40% after 7 min. The column was flushed with 40% B for 1 min. After regeneration of the starting conditions for 0.5 min, the column was re-equilibrated at 100% A for three additional minutes.

### Calibration for absolute quantification of modified nucleosides

For calibration, synthetic nucleosides were weighed and dissolved in water to a stock concentration of 1–10 mM. The calibration solutions range from 0.3 to 500 pmol for each canonical nucleoside and from 0.3 to 500 fmol for each modified nucleoside and were spiked with 10% SILIS according to Borland *et al.* (53). Data were analyzed using the Quantitative and Qualitative MassHunter Software from Agilent.

### Analysis of tRNA stability and aminoacylation

*In vivo* RNA stability was analyzed by cell treatment with transcription inhibitors (54). *Escherichia coli* strains were cultivated in 500 ml LB medium (Lennox) to an OD_600_ of 0.5 before addition of 500 μg/ml rifampicin (stock solution 100 mg/ml in DMSO) and 40 μg/ml nalidixic acid (stock solution 25 mg/ml, titrated with 1 M NaOH until solubility). Directly after addition (*t*0) and at several time points, 50 ml of the cell cultures were harvested for total RNA isolation. 12 μg total RNA from each sample were separated on a 10% denaturing acrylamide gel with 5 M urea (20 × 20 cm) in 1× TBE for 3 h at 16 W. RNAs were transferred to a Hybond N+ membrane by tank blotting for 18 h at 15 V. Oligonucleotide tArg-8651 was radiolabeled with T4 polynucleotide kinase using γ-[^32^P]-ATP and hybridized to the membrane. Signals were detected by phosphoimaging using a Typhoon 9100 (GE Healthcare).

For analysis of tRNA aminoacylation, total RNA was isolated under acidic conditions as described by Varshney *et al.* (55). After precipitation, RNAs were redissolved in 10 mM NaOAc pH 4.5 or, as control, in 0.1 M Tris–HCl, 1 mM EDTA pH 9.0 followed by a deaminoacylation for 30 min at 37°C. 1 μg of each RNA sample were mixed with an acidic sample buffer (0.1 M NaOAc pH 5.0, 8 M urea, 0.05% bromphenol blue, 0.05% xylene cyanol) and denatured for 3 min at 95°C. Acidic acrylamide gels (20 × 20 cm) were prepared as described before (56) and RNA samples were separated for 17 h with 100 V at room temperature. Northern blotting and detection of tRNA^Arg^-ICG was performed as described above.

## RESULTS AND DISCUSSION

### E. *coli* YfiP is a bacterial homolog of the eukaryotic rRNA acp transferase Tsr3

**Figure 2.**
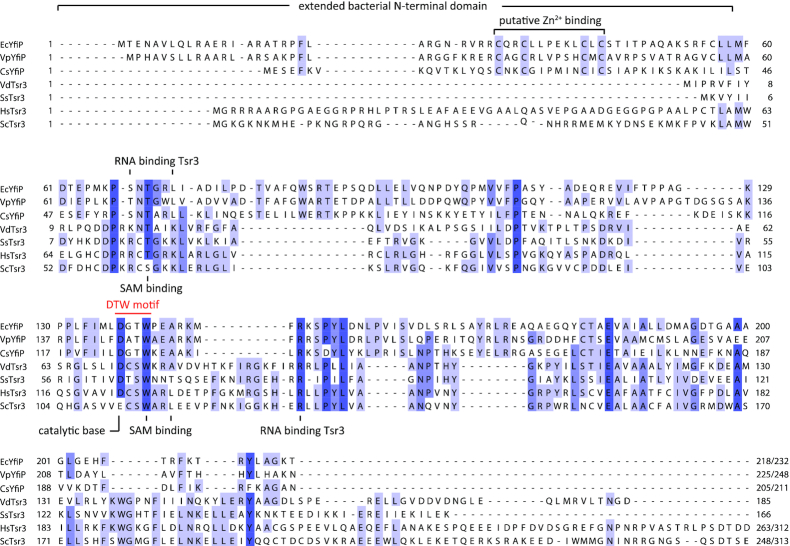
Clustal Omega alignment of eukaryotic and archaeal Tsr3 proteins and bacterial YfiP homologs with the DTW domain and the conserved cysteines predicted to bind Zn^2+^. Ec: *Escherichia coli*; Vp: *Variovorax paradoxus*; Cs: *Clostridium saccharoperbutylacetonicum*; Vd: *Vulcanisaeta distributa*; Ss: *Sulfolobus solfataricus*; Hs: *Homo sapiens;* Sc: *Saccharomyces cerevisiae*.

Sequence alignments between eukaryotic and in particular archaeal Tsr3 homologs and bacterial YfiP-like proteins reveal that the sequence homologies are restricted to the catalytic core domain of Tsr3-like proteins (Figure 2). In this core region, many functionally important residues are strictly conserved as expected from proteins with a similar catalytic function. Besides the amino acids in the DTW motif this includes other amino acids such as T19 and A76 (*V. distributa* numbering, Figure 2) directly involved in SAM binding. Interestingly, basic amino acids shown to be important for target RNA binding in Tsr3 proteins are not conserved in *E. coli* YfiP possibly pointing toward a different mode of target RNA recognition. Compared to the archaeal Tsr3-like proteins *E. coli* YfiP and its bacterial homologs have a significantly extended N-terminal region (∼70 aa). However, the sequence of this N-terminal extension shows only conservation between bacterial homologs but not with those of the N-terminal extensions of eukaryotic Tsr3-homologs which have been shown experimentally to be functionally unimportant (33). Thus, bacterial YfiP-like proteins apparently belong to a subclass of DTW-domain containing proteins with a function different from the Tsr3-like proteins in agreement with previous bioinformatics results (39). Particularly noteworthy features of the N-terminal extension of YfiP-like proteins are the presence of four highly conserved cysteine residues and its highly overall basic character (p*I* ∼ 11). The four cysteine residues which are organized in a CXXCXXXXXXCXC motif could either form the binding site for an iron–sulfur cluster or a zinc binding site typical of zinc finger, zinc knuckle or zinc ribbon domains. Zinc binding domains are frequently observed in RNA-binding proteins. This would be in line with the proposed function of YfiP as an RNA modification enzyme able to recognize a different set of RNA substrates compared to the rRNA-modifying Tsr3 homologs. Zinc binding domains as RNA recognition elements have been previously found in other RNA methyltransferases such as METTL3 and Trm13 (57,58) or in the Trm112 scaffolding protein that forms complexes with a variety of RNA modification enzymes (59,60). Examples for YfiP-like proteins can be found in many bacterial lineages. They are common but not ubiquitous in the genomes of enterobacteriacea in particular and in the γ-proteobacteria in general but also occur in other lineages of the proteobacteria. They also appear with low frequency in the genomes of Gram-positive bacteria e.g. in some firmicutes.

### A yfiP knock-out abrogates the acp^3^U modification in *E. coli* tRNA

**Figure 3.**
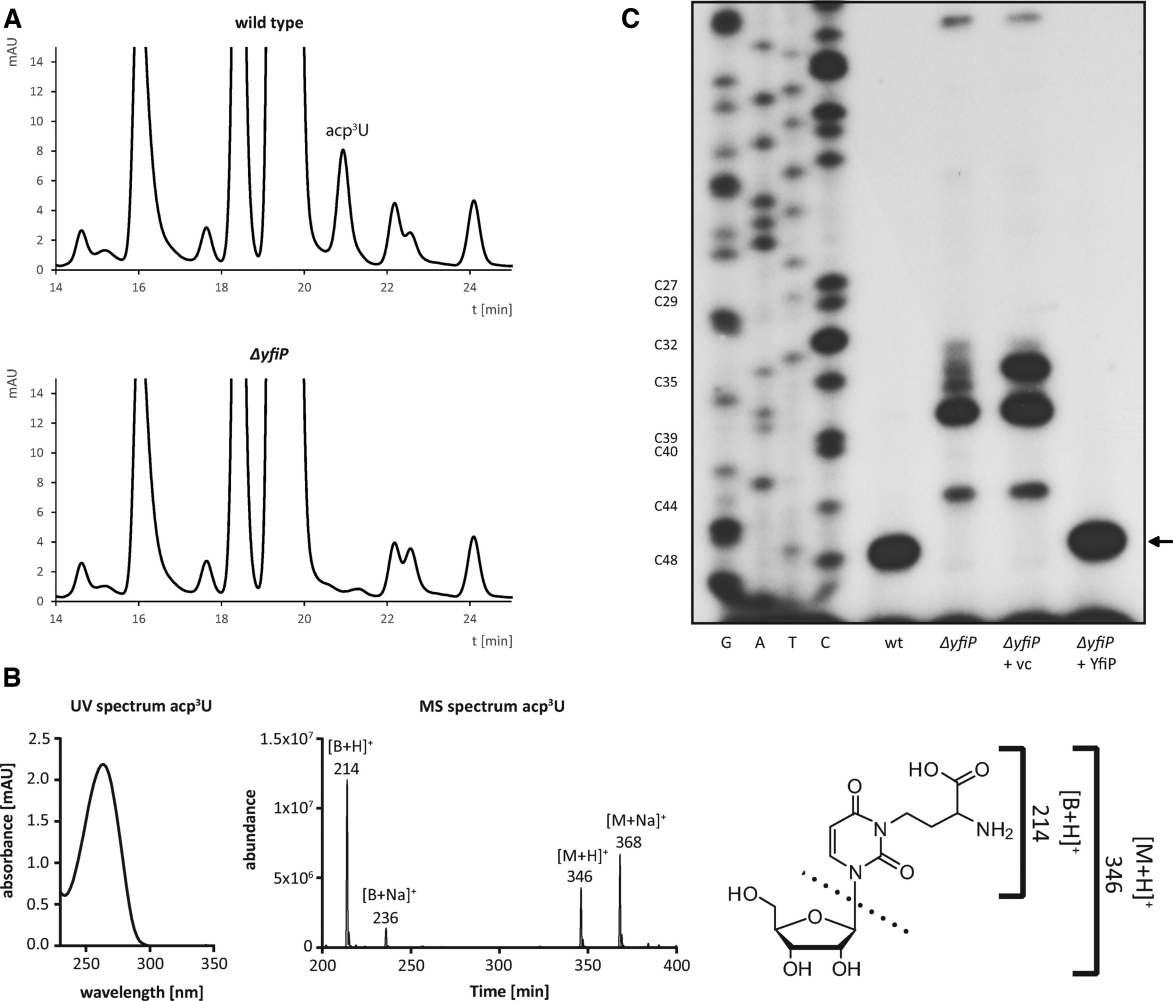
YfiP is necessary for *E. coli* tRNA acp modification. (**A**) RP-HPLC elution profiles of tRNA nucleosides from wild type and *ΔyfiP E. coli* cells. acp^3^U elutes at 20.9 min and is missing in total tRNA from the *ΔyfiP* strain. (**B**) UV and MS spectra of purified acp^3^U. Left: UV spectrum of acp^3^U in 5 mM NH_4_OAc (pH 5.3) as detected in the diode array detector coupled to LC–MS/MS. Right: MS spectrum of acp^3^U at 180 V fragmentor voltage. (**C**) Primer extension analysis of tRNA^Arg^-ICG acp modification including a sequencing ladder. The reverse transcriptase arrest at C48 is missing in the *ΔyfiP* strain and can be restored by complementation with plasmid encoded YfiP (*ΔyfiP* + YfiP; vc: empty vector control).

A *ΔyfiP E. coli* deletion strain was constructed by the one-step inactivation method using the λ-Red recombination system (42). The disruption of the *yfiP* gene by a FRT-kanR-FRT deletion cassette and subsequent removal of the antibiotic resistance gene after expressing the Flp recombinase was confirmed by diagnostic PCR. WT and *ΔyfiP E. coli* cells were grown in LB medium to an optical density of 1, cells were harvested and the small RNAs including tRNAs of both strains were isolated from total RNA by sucrose gradient centrifugation. 60 μg of these RNAs were hydrolyzed and dephosphorylated by treatment with nuclease P1 and bacterial alkaline phosphatase (46) and the resulting nucleosides were separated by reversed phase HPLC using well-established methods (47). A peak corresponding to a nucleoside eluting at 20.9 min was missing specifically in the elution profile from the Δ*yfiP* cells as compared to wild type cells (Figure 3A). Its retention time and its elution directly after the major nucleoside G were very similar to the elution conditions previously reported for acp^3^U (46). Electrospray ionization mass spectrometry (ESI-MS) provided precursor ions of 346 [M+H]^+^ and 368 [M+Na]^+^ for this nucleoside as well as 214 [B+H]+ and 236 [B+Na]^+^ for the respective product ions. These m/z values are in agreement with the theoretical acp^3^U mass of 345.11 confirming that the missing peak in the HPLC elution profile of the Δ*yfiP* cells indeed corresponds to acp^3^U (Figure 3B). Thus, a *yfiP* deletion in *E. coli* leads to an apparently complete loss of the acp^3^U modification in *E. coli* tRNAs.

As shown for yeast 18S rRNA, m^1^acp^3^Ψ as well as acp^3^U modifications strongly block reverse transcriptase activity (33,61,62), and thus in a primer extension analysis a strong stop at the position preceding the acp-modified site is expected. Therefore, tRNA samples from wild type and *ΔyfiP* cells were initially subjected to primer extension analysis using oligonucleotide Arg-8651 (Supplementary Table S4). Arg-8651 is complementary to the C-terminal part of tRNA^Arg^-ICG which contains an acp^3^U modification at position 47 and is highly abundant in *E. coli*. As expected, a strong primer extension stop signal was observed at position C48 in wild type tRNA, but not in tRNA from *E. coli ΔyfiP* cells. The acp^3^U-47 modification was restored upon transformation of the *ΔyfiP* strain with YfiP encoded on plasmid pPK895 behind the T5-lac promoter, but not upon transformation with the empty vector (Figure 3C).

**Figure 4.**
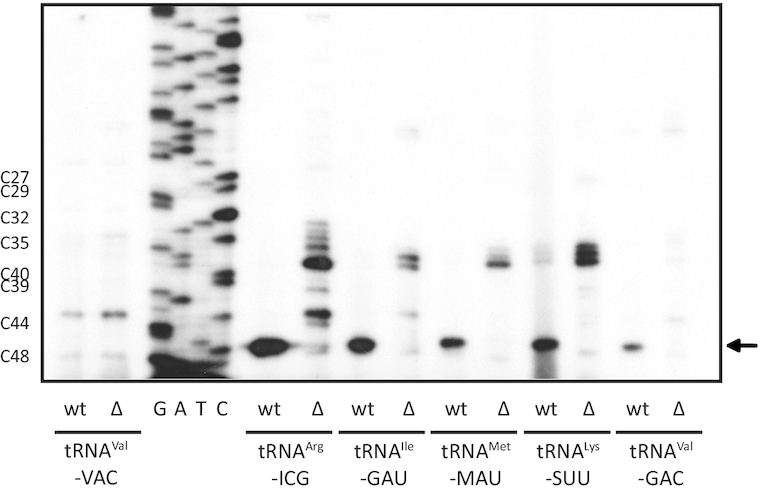
Primer extension analysis of acp^3^U-47 modification of different putative target tRNAs using specific oligonucleotides hybridized to tRNA samples from wild type (wt) and *ΔyfiP* (Δ) *E. coli* cells. YfiP depending arrest signals of varying intensities are detectable for five different tRNAs, but not for the control tRNA^Val^-VAC carrying no acp^3^U-47.

The acp^3^U modification at U47 was described to occur in 8 different *E. coli* tRNAs from 6 different isotypes (Figure 1B, Supplementary Figure S1, Table S1). In addition to tRNA^Arg^-ICG, primer extension experiments using the appropriate complementary oligonucleotides were performed for four additional tRNAs known to carry the acp^3^U modification at U47 (Supplementary Figure S1) as well as for tRNA^Val^-VAC (V = uridine 5-oxyacetic acid, cmo^5^U) which is not acp modified at U47. As expected, no primer extension stop signal at nucleotide 48 occurred in case of the negative control tRNA^Val^-VAC (Figure 4), demonstrating the specificity of the detection method. However, primer extension stops were clearly observable for tRNA^Arg^-ICG, tRNA^Ile^-GAU, tRNA^Met^-MAU, tRNA^Lys^-SUU and tRNA^Val^-GAC from wild type cells (Figure 4). In all these cases, the primer extension stops were missing in the *ΔyfiP* cells (Figure 4), indicating that YfiP is involved in the biosynthesis of the acp^3^U modification at position 47 for all examined tRNAs. Although equal amounts of total tRNAs were applied in all primer extension experiments, different primer extension oligonucleotides yielded stop signals of different intensities for the individual tRNAs (Figure 4). This might be due to unequal levels of specific template tRNAs, varying binding affinities of the respective oligonucleotides, or could be even due to incomplete acp modification in certain tRNA species under the assessed conditions. However, in the latter case this should lead to primer extension stop signals at different positions due to the presence of modified nucleotides preceding position 47 which are not observable for the five tRNAs tested.

### *In vitro* activity of the *E. coli* YfiP protein

In order to characterize the activity of the YfiP protein *in vitro*, it was overexpressed in *E. coli* as a construct with a C-terminal His-tag and purified to homogeneity by a combination of affinity chromatography, cation exchange chromatography and gel filtration (Supplementary Figure S2). Analytical gel filtration revealed that the protein is a monomer in solution (Supplementary Figure S2) similar to our previous findings for the rRNA acp-transferase Tsr3 (33) but in contrast to many other SPOUT-class proteins which usually form stable dimers. The purified homogeneous protein is colorless in solution and shows no absorption at wavelengths >300 nm. This demonstrates the absence of a stably bound iron-sulfur cluster. The purified protein contains no bound SAM as indicated by the *A*_260_/*A*_280_ ratio in the UV/Vis spectrum (Supplementary Figure S2). However, the purified *E. coli* YfiP contains ∼1 stably bound zinc ion per protein molecule according to our ICP-MS analysis (Supplementary Table S5). Furthermore, a distant sequentially homologous protein from the Gram-positive firmicute *Clostridium saccharoperbutylacetonicum* (see below) also binds ∼1 zinc ion per protein molecule. Thus, YfiP homologs are apparently genuine zinc-binding proteins.

**Figure 5.**
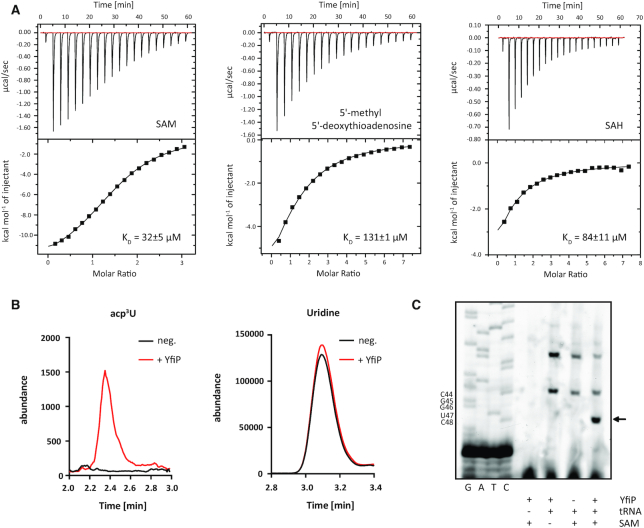
*In vitro* activity of the *E. coli* YfiP protein. (**A**) Representative ITC thermograms for titrations of purified YfiP protein with either SAM (left), 5-MTA (center) or SAH (right). The resulting dissociation constants are indicated. The cofactor SAM is bound with the highest affinity. (**B**) Abundance of acp^3^U after *in vitro* modification of total tRNA isolated from a *ΔyfiP* strain with overexpressed and purified YfiP. Equal amounts of total tRNA from mock-incubated (black) and YfiP incubated samples (red) were analyzed by nucleoside LC-MS/MS. On the left the abundance of acp^3^U (mass transition 346 → 214) is shown and on the right uridine (mass transition 245 → 113) is shown. (**C**) Detection of acp^3^U-47 in tRNA^Arg^-ICG by a primer extension stop at position C48 after *in vitro* incubation of total tRNA from a *ΔyfiP* strain with purified YfiP and SAM. The reverse transcriptase arrest at C48 is missing if the protein or the cofactor SAM are excluded from the reaction.

Isothermal titration calorimetry showed that the purified protein bound to SAM with a dissociation constant *K*_D_ of 32 μM and with a reduced affinity to the putative product of a SAM-dependent acp-transfer reaction 5′-methyl 5′-deoxythioadenosine (5-MTA) (*K*_D_ = 131 μM) in agreement with its putative function as a SAM-dependent acp transferase (Figure 5A). The affinity of YfiP for *S*-adenosylhomocysteine (SAH) is also considerably lower (84 μM) than its affinity for SAM. The measured dissociation constants are in a range typical for many SAM-binding RNA methyltransferases and comparable to other acp transferases such as Tsr3 (33).

In order to test for an acp transferase activity of purified YfiP *in vitro*, purified total tRNA from the *E. coli ΔyfiP* strain which according to HPLC analysis does not contain acp^3^U was incubated with SAM and overexpressed and purified *E. coli* YfiP protein. LC-MS analysis of the total tRNA before and after incubation with YfiP showed that in the presence of SAM and YfiP acp^3^U is introduced into the tRNA whereas no acp^3^U is present in total tRNA after the incubation if the protein was not included in the reaction (Figure 5B).

Similarly, primer extension experiments with tRNA^Arg^-ICG before and after incubation of purified total tRNA from the *E. coli ΔyfiP* strain with purified YfiP and SAM *in vitro* showed that a primer extension signal at C48 only occurs after incubation with the protein and the cofactor but not in the absence of one or the other or both (Figure 5C). Thus, together with our *in vivo* data these experiments show that the YfiP protein is necessary and sufficient to introduce the acp^3^U modification at position U47 in *E. coli* tRNAs and is therefore the up-to-now undescribed acp transferase enzyme.

### Determinants of acp transferase activity in *E. coli* YfiP

**Figure 6.**
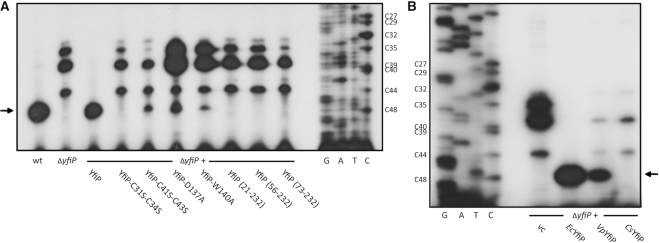
Determinants of acp transferase activity in *E. coli* YfiP. (**A**) Truncated or point mutated YfiP variants were expressed in *E. coli ΔyfiP* cells, and the degree of acp^3^U-47 modification of tRNA^Arg^-ICG was analyzed by primer extension in comparison to the wild type (wt) and *ΔyfiP* cells with the empty vector (vc). (**B**) As shown by primer extension analysis, the YfiP homolog from *Variovorax paradoxum* (*Vp*), but not from *Clostridium saccharoperbutylacetonicum* (*Cs*), is functional as an acp^3^U-47 transferase in *E. coli ΔyfiP*.

Compared to the archaeal and eukaryotic small ribosomal subunit rRNA acp-transferase Tsr3 the YfiP protein contains an extended basic N-terminus of ∼ 70 aa that contains four conserved cysteine residues in a CXXCXXXXXXCXC motif. This N-terminal region apparently forms an N-terminal zinc binding domain with a putative function in tRNA recognition. To test the functional importance of this region *in vivo*, we overexpressed N-terminal truncation mutants of *E. coli* YfiP in the *ΔyfiP* strain and analyzed their influence on acp^3^U modification of U47 in tRNA^Arg^-ICG in primer extension experiments. N-terminal deletions of 20, 55 or 72 amino acids, the last two including the conserved cysteine residues, completely inhibited the acp^3^U-47 modification of endogenous tRNA^Arg^-ICG (Figure 6A). Similarly, the combined exchange of cysteines C31 and C34 to serine in the context of the full-length protein also completely blocked the acp modification, whereas a C41S-C43S double mutant showed a weak residual activity (Figure 6A). Taken together, this argues for an important functional role of the N-terminal domain and in particular its zinc-binding motif for acp transferase activity.

In their SAM-binding catalytic core domain all DTW-containing proteins contain a D/E-X-S/T-W consensus motif. The residue conserved as D/E in the motif is probably the catalytic base deprotonating the U nucleotide prior to the acp transfer. Mutation of the corresponding D137 to A in *E. coli* Yfip strongly diminished acp transfer activity in agreement with such a function and with previous results for yeast Tsr3 (33). Structural analysis of the archaeal Tsr3 protein from *Vulcanisaeta distributa* showed that the conserved W of the motif (W73 in *Vulcanisaeta distributa* Tsr3, W114 in *S. cerevisiae* Tsr3, Figure 2) is directly involved in binding of the cofactor SAM in a binding mode enabling the specific transfer of the acp-group of SAM. A mutation of W114 in yeast Tsr3 to alanine strongly reduces its *in vivo* acp transferase activity (33). Similarly, the corresponding W140A mutation in *E. coli* YfiP lead to a strongly reduced intensity of the primer extension stop signal for tRNA^Arg^-ICG suggesting a strongly reduced level for the acp^3^U-47 modification in the presence of the mutated protein (Figure 6A) and a similar function of W140 in SAM binding compared to Tsr3.

Finally, we tested the ability of two distant relatives of *E. coli* YfiP to functionally replace YfiP *in vivo* as the acp transferase for U47 using primer extensions experiments with tRNA^Arg^-ICG. When the YfiP homolog from the Gram-negative β-proteobacterium *Variovorax paradoxum* is overexpressed in an *E. coli ΔyfiP* strain a primer extension stop corresponding to the acp modification at U47 is still observable albeit with a reduced intensity suggesting that this protein is also a functional homolog of *E. coli* YfiP. No primer extension stop is observed upon overexpression of the YfiP homolog from the Gram-positive firmicute *Clostridium saccharoperbutylacetonicum* under the same conditions suggesting that this protein cannot functionally replace *E. coli* YfiP (Figure 6B). In principle, the inactivity of the YfiP homolog from *C. saccharoperbutylacetonicum* in *E. coli* cells could be due to either insufficient expression of this distant homolog or the incomplete folding of the heterologously expressed protein. However, heterologous expression of the YfiP homolog from *C. saccharoperbutylacetonicum* in *E. coli* cells and subsequent purification yielded a well-folded protein according to its CD-spectrum (Supplementary Figure S3). Thus, the lack of activity of the *C. saccharoperbutylacetonicum* YfiP homolog in *E. coli* is more likely caused by differences in the modification patterns or structure of the tRNA targets in the two organisms that prevent the proper recognition of *E. coli* tRNA^Arg^-ICG. Unfortunately, nothing is known about tRNA modifications in *C. saccharoperbutylacetonicum*.

### The TrmB catalyzed methylation of G46 to m^7^G promotes acp^3^U formation at U47

In all tRNAs containing acp^3^U at position 47, the preceding nucleotide is methylated to m^7^G-46 (Figure 1). However, the m^7^G modification at position 46 is also present in a number of other tRNAs that do not carry an acp^3^U modification at position 47. The gene encoding the enzyme responsible for the introduction of the m^7^G modification at position 46 in *E. coli* was originally known as *yggH* (63). According to the EcoCyc database, the corresponding enzyme should be named TrmI. Unfortunately, in *Thermus thermophilus* and in many other bacteria TrmI is used as the name for a tRNA methyltransferase modifying the N1 nitrogen of A58 (64). Furthermore, the tRNA m^7^G-46 methyltransferase is named TrmB in all other bacteria were this enzyme was investigated and this name is also used frequently in reference to the *E. coli* homolog. Therefore, we use the name TrmB for the *E. coli* enzyme responsible for introducing the m^7^G modification at position 46 in this work. *E. coli* TrmB is not an essential protein and no phenotype was reported for the *ΔyggH* knock-out strain (63). Interestingly, for *T. thermophilus* tRNAs it was shown that the loss of the m^7^G modification at position 46 lead to a partial loss of modifications in other positions (65). Thus, the presence of m^7^G at position 46 promotes the biosynthesis of other modifications suggesting the presence of networks of interdependent modifications in tRNAs. However, the acp^3^U modification is not present in *T. thermophilus* tRNAs.

**Figure 7.**
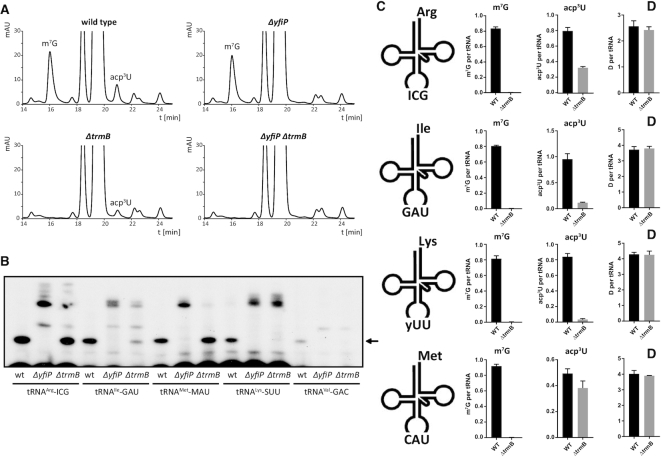
Dependency of the acp^3^U-47 modification on the presence of the m^7^G-46 methyltransferase TrmB. (**A**) HPLC chromatograms of nucleosides in total tRNA from single and double deletion strains compared to the *E. coli* wild type. The amount of acp^3^U is significantly reduced in the *ΔtrmB* mutant, whereas a *ΔyfiP* deletion has no influence on m^7^G formation. (**B**) Primer extension analysis comparing the level of the acp^3^U-47 modification in wild type and *ΔyfiP* or *ΔtrmB* deleted cells. The acp^3^U dependent primer extension stop signal is strongly reduced by the *ΔtrmB* deletion for tRNA^Ile^-GAU, tRNA^Lys^-SUU and tRNA^Val^-GAC. (**C**) Absolute quantification of modified nucleosides in purified tRNA isoacceptors. *E. coli* wild type (WT) and Δ*trmB* were grown in LB medium, total tRNA was isolated and tRNA isoacceptors were purified as previously published (52). Absolute quantification of modified nucleosides was performed by LC–MS/MS using stable isotope labeled internal standards following published protocols (53). D, dihydrouridine (*n* = 5).

To analyze a putative mutual dependence of the m^7^G and acp^3^U modifications at positions 46 and 47 from each other, tRNA modification profiles were analyzed on the nucleoside level by HPLC using total tRNA from wild type as well as from single (*ΔyfiP* or *ΔtrmB*) and double deletion mutant (*ΔyfiP ΔtrmB*) strains. Whereas the relative amount of m^7^G was similar for the *ΔyfiP* mutant and the wild type strain, the acp^3^U HPLC signal was significantly decreased in the *ΔtrmB* mutant (Figure 7A). The amount of acp^3^U was reduced to ∼40% in the *ΔtrmB* strain compared to the wild type based on a quantitative comparison of HPLC chromatograms (Supplementary Figure S4 and Table S6). As expected, both acp^3^U and m^7^G nucleosides are completely missing in the double deletion strain *ΔyfiP ΔtrmB* (Figure 7A). Thus, the presence of the m^7^G modification at position 46 apparently increases the effectiveness of the acp^3^U modification at position 47. To analyze the influence of the m^7^G mutation on the presence of acp^3^U at position 47 on the level of individual tRNAs we carried out primer extension analysis with individual oligonucleotides specific for five different tRNAs and compared the intensity of primer extension stop signals in tRNAs isolated from wild type, *ΔyfiP* and *ΔtrmB* strains. Remarkably, the influence of the TrmB deletion on the intensity of the acp dependent stop signals varied for individual tRNAs (Figure 7B). The primer extension stop signal corresponding to the U47 modification in tRNA^Arg^-ICG and tRNA^Met^-MAU was strong in both the wild type and in the *ΔtrmB* mutant suggesting that here the influence of the TrmB deletion on the modification of U47 is small. However, even for these tRNAs additional primer extension stop signals become visible for nucleotides preceding position 47 which are not observable in the wild type suggesting the presence of tRNAs carrying an unmodified U47 in the *ΔtrmB* deletion strain. In contrast, the primer extension stop signals corresponding to the acp^3^U modification of tRNA^Ile^-GAU, tRNA^Lys^-SUU and tRNA^Val^-GAC were strongly diminished by the *ΔtrmB* deletion in comparison to the wild type (Figure 7B). Thus, for these tRNAs the presence of the m^7^G modification at position 46 strongly promotes the introduction of the acp^3^U modification at position 47. In order to quantify these effects, we resorted to absolute quantification of modified nucleotides in isolated tRNA isoacceptors by isotope dilution mass spectrometry (53). The results showed a substantially decreased abundance of acp^3^U in the Δ*trmB* mutant for tRNA^Ile^-GAU and tRNA^Lys^-SUU but a substantially smaller decrease in acp^3^U abundance for tRNA^Arg^-ICG and tRNA^Met^-MAU (Figure 7C). Thus, the m^7^G and acp^3^U modifications at positions 46 and 47 in the variable loop present an additional example for interdependent modifications in a modification network. However, sequence and structural differences between different tRNA species apparently modulate the strength of the coupling between these two modifications. Furthermore, a clear hierarchy for the two modifications is present – the absence of m^7^G reduces the efficiency of the acp^3^U modification but the absence of the acp^3^U modification does not influence the efficiency of the m^7^G modification.

### Medium composition modulates the degree of acp^3^U modification

**Figure 8.**
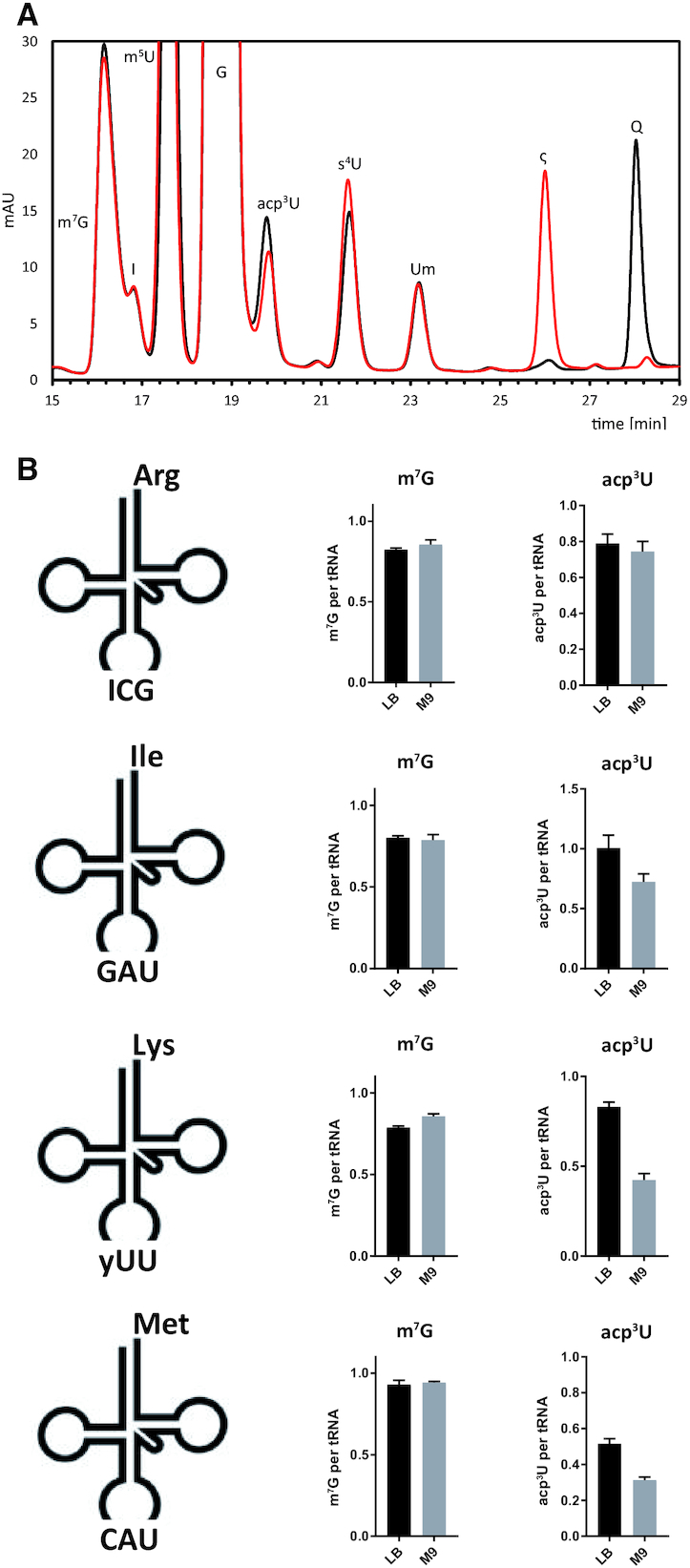
Influence of the growth medium on the level of acp^3^U modification. (**A**) RP-HPLC elution profiles of tRNA nucleosides from wild type *E. coli* cells cultivated in LB (black) or M9 (red) medium. Q, queuosine; ς, epoxyqueuosine. (**B**) Absolute quantification by LC–MS/MS of modified nucleosides in purified tRNA isoacceptors from wild type cells grown in LB and M9 medium at OD 0.4 (*n* = 3).

The presence of some chemically complex modifications in bacterial tRNAs is influenced by medium composition (66,67). During the formation of acp^3^U, SAM is consumed which is energetically costly to synthesize from an ATP precursor. We therefore analyzed the influence of the growth medium on the degree of the acp^3^U modification by comparing the levels of acp^3^U for WT *E. coli* grown either in LB or in minimal mineral medium (M9) with glucose and ammonium chloride as the only available carbon and nitrogen sources. Quantitative analysis of acp^3^U levels in total tRNA from cells grown in both media by HPLC showed acp^3^U levels reduced by ∼20% upon growth in M9 medium (Figure 8A, Supplementary Figure S5, Table S6). Another modification strongly affected by growth on different media was queuosine (Q). The HPLC signal corresponding to queuosine is much higher for cells grown in LB medium compared to cells from M9 medium. In contrast, for cells grown in M9 medium the queuosine precursor epoxyqueuosine (ς) is much more abundant due to the lack of vitamin B_12_ in M9 medium in agreement with previous results in *S. typhimurium* (66). For absolute quantification on the level of individual tRNAs, we applied again isotope dilution mass spectrometry of isolated tRNAs. Indeed, we observe a significantly lower abundance of acp^3^U in tRNA^Lys^-SUU, tRNA^Ile^-GAU and tRNA^Met^-CAU but not in tRNA^Arg^-ICG while the abundance of m^7^G remained unchanged (Figure 8B).

### Phenotypic analysis of the acp transferase YfiP deletion in *E. coli*

**Figure 9.**
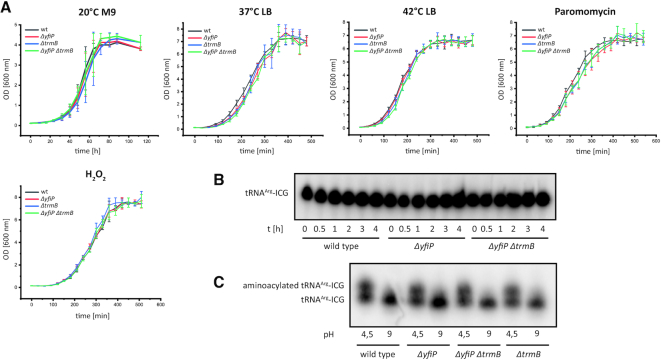
Phenotypic analysis of deletion mutants lacking variable loop modifications. (**A**) Growth curves (*n* = 3) for *E. coli ΔyfiP* lacking acp^3^U-47, *ΔtrmB* lacking m^7^G-46 and *ΔyfiP ΔtrmB* lacking both modifications in comparison to the wild type strain at 20°C in M9 minimal medium, at 37°C and 42°C in LB medium and in the presence of paromomycin (2.5 μg/ml) or 10 mM H_2_O_2_. The deletion mutants do not show differences in growth rate compared to the wild type under all conditions tested. (**B**) The stability of the tRNA^Arg^-ICG from *E. coli* wild type, the *ΔyfiP* and the *ΔyfiP ΔtrmB* deletion strains was analyzed by northern blotting after cultivation with the transcription inhibitors rifampicin and nalidixic acid for the indicated amounts of time (hours). The comparison of the tRNA^Arg^-ICG amounts present at equivalent time points in the different strains shows that the relative stability of this tRNA is similar in all tested strains. (**C**) In order to compare the aminoacylation efficiency for modified and unmodified tRNA^Arg^-ICG, total RNAs of the wild type and the *ΔyfiP* strains were isolated and gel-separated under acidic conditions (pH 4.5), and aminoacylated and deaminoacylated tRNA^Arg^-ICG was detected by northern blotting. A part of each RNA sample was incubated separately with buffer at pH 9.0 for complete deaminoacylation.

The genes encoding enzymes catalyzing modifications in the body of tRNAs are often not essential and their deletion frequently does not lead to observable phenotypes (68–70). We nevertheless were interested in the consequences of a YfiP deletion and a YfiP/TrmB double deletion resulting in a completely unmodified variable loop on the growth rate and stress sensitivity of *E. coli*. A TrmB deletion in *E. coli* showed that this gene was not essential for growth and no phenotype was reported (63). However, a deletion of the gene encoding the TrmB homolog in *Pseudomonas aeruginosa* induced an increased sensitivity towards H_2_O_2_ (71). Furthermore, a TrmB deletion in *T. thermophilus* lead to growth retardation and diminished protein synthesis at elevated temperatures, increased degradation of some tRNA species and a decrease in tRNA melting temperature (65). We cultivated *E. coli ΔyfiP* lacking acp^3^U-47 and *ΔtrmB* (lacking m^7^G-46) strains as well as the double deletion mutant *ΔyfiP ΔtrmB* under varying conditions with regard to growth media, temperature and different stress conditions. Growth curves for all deletion mutants recorded in M9 minimal or LB medium at different temperatures (20, 37 and 42°C) showed no clear differences in comparison to the wild type strain (Figure 9A). Furthermore, in the presence of different stress factors such as H_2_O_2_ (oxidative stress; 10 mM) or the translational inhibitor paromomycin (sublethal concentrations up to 2.5 μg/ml) the growth curves of all deletion strains were similar to those of the wild type strain (Figure 9A). This indicates a negligible influence of acp^3^U-47 and m^7^G-46 on the proper function of the respective tRNAs *in vivo*.

Furthermore, the influence of YfiP and TrmB catalyzed modifications on tRNA stability and aminoacylation was assessed. To this end, *E. coli* wildtype, *ΔyfiP* and *ΔyfiP ΔtrmB* strains were cultivated in the presence of the transcription inhibitors rifampicin and nalidixic acid to investigate the stability of one of the acp^3^U modified tRNAs (tRNA^Arg^-ICG). At different time points after addition of the transcription inhibitors, cells were harvested, and total RNA was isolated from the different strains. As a result of the incubation with the transcription inhibitors, the overall amount of isolated RNA per OD_600_ unit decreased to about half after two hours as expected. Constant amounts of total RNA from each time point and strain were then separated by denaturing PAGE. tRNA^Arg^-ICG was detected by northern blot analysis (Figure 9B, Supplementary Figure S6). The relative amounts of tRNA^Arg^-ICG present at the same time points after transcription inhibition are very similar between the wild type and the different deletion strains. Thus, the stability of tRNA^Arg^-ICG does not differ between wild type and *ΔyfiP*, or *ΔyfiP ΔtrmB* cells (Figure 9B, Supplementary Figure S6), arguing against a severe influence of acp^3^U-47 or m^7^G-46 on the stability of tRNA^Arg^-ICG.

*In vivo* aminoacylation of tRNA^Arg^-ICG was analyzed by acidic denaturing gel electrophoresis and Northern blotting (55,56). Total RNA from the different *E. coli* strains was isolated under acidic conditions. As a control, the tRNA was deaminoacylated by incubation at pH 9.0 in an aliquot of each RNA sample. The *ΔyfiP* or *ΔtrmB* deletions strains as well as the double mutant *ΔyfiP ΔtrmB* showed a similar ratio between aminoacylated and deaminoacylated tRNA^Arg^-ICG as compared to that of the wild type (Figure 9C). Thus, the biological function of the acp^3^U modification of position 47 in *E. coli* tRNAs currently remains unclear. Further analysis including a wider variety of stress conditions, the characterization of the translational efficiency on the level of individual mRNA transcripts containing codons read by acp^3^U containing tRNAs as well as translatome-wide approaches will probably be required to better define the function of the modifications at position 46 and 47 in *E. coli* RNAs. Experiments in this direction are currently in progress in our laboratories.

Overall, in our work presented here we demonstrate that the so far functionally uncharacterized *E. coli* protein YfiP is the enzymatic factor necessary and sufficient to introduce the acp^3^U modification at position U47 in *E. coli* tRNAs. We therefore rename this protein **t**RNA **U**47 **a**minocarboxypropyl transferase **A** or TuaA. Thus, *E. coli* is now the first organism where all tRNA modifications described so far have been connected to their corresponding modification enzymes. Interestingly, the degree of acp^3^U modification at position 47 depends on the prior presence of the m^7^G modification at the preceding nucleotide 46 in the variable loop. However, this dependence is more pronounced for some of the acp^3^U-47 modified tRNAs than for others. The degree of the acp^3^U-47 modification also depends on medium composition. In minimal mineral medium with only ammonium chloride and glucose as the sole nitrogen and carbon sources, respectively, this position is not completely modified. The biological function of the acp^3^U modification at position 47 could not yet be established.

When taken together with our previous work regarding the identification of Tsr3 as the acp transferase enzyme responsible for modifying U or m^1^Ψ in archaeal and eukaryotic rRNAs and its structural characterization the results presented here suggest that many if not all DTW domain containing proteins have a SAM-dependent acp transferase activity. Thus, our results should be helpful in guiding the discovery of e.g. the eukaryotic DTW-domain containing proteins responsible for introducing the acp^3^U modification, e.g. in position 20a in mammalian tRNAs.

## Supplementary Material

gkz1191_Supplemental_File
