# Supplementary material for: Identification of the 3-amino-3-carboxypropyl (acp) transferase enzyme responsible for acp3U formation at position 47 in Escherichia coli tRNAs
Source: Nucleic Acids Res. 2019 Dec 21;48(3):1435–50. doi: 10.1093/nar/gkz1191 (PMC7026641; doi:10.1093/nar/gkz1191)
Supplement: gkz1191_Supplemental_File [file gkz1191_supplemental_file.pdf]

Supplementary Data

Identification of the 3-amino-3-carboxypropyl (acp) transferase enzyme responsible for acp^{3}U formation at position 47 in *Escherichia coli*

Britta Meyer, Carina Immer, Steffen Kaiser, Sunny Sharma, Jun Yang, Peter Watzinger, Lena Weiß, Annika Kotter, Mark Helm, Hans-Michael Seitz, Peter Kötter, Stefanie Kellner, Karl-Dieter Entian, Jens Wöhnert

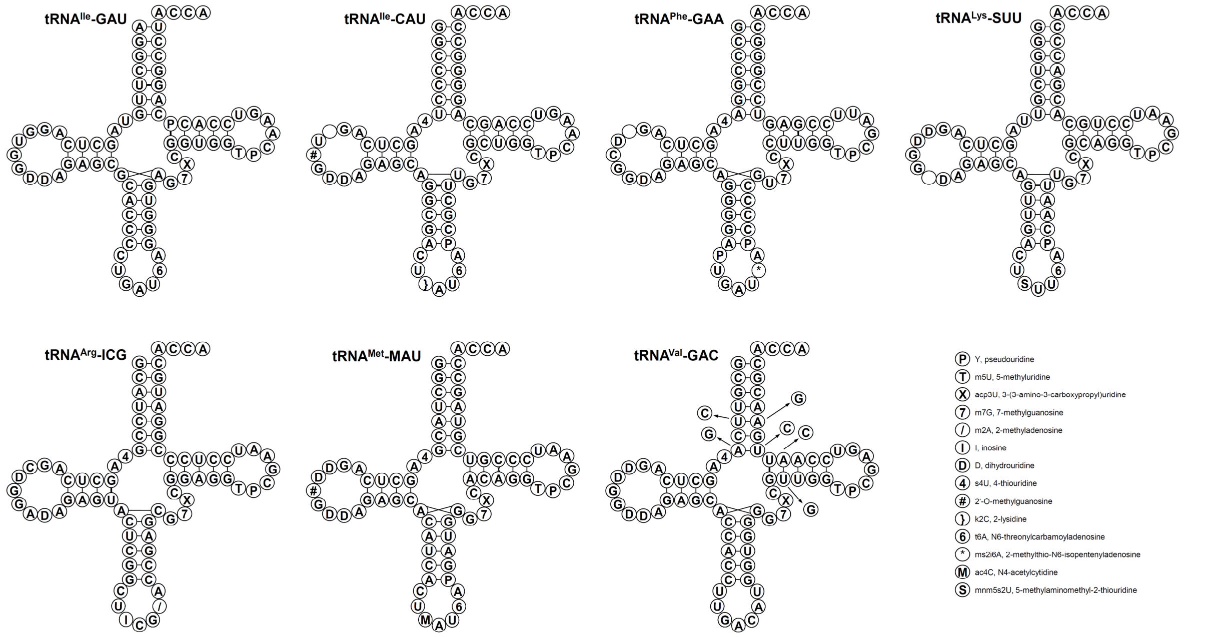

Supplementary Figure S1: Predicted secondary structures of *E. coli* tRNA isosacceptors with an acp^{3}U (X) modification at position 47. Two tRNA genes (*valV* and *valW*) coding for the tRNA^{Val}-GAC exist in the genome of *E. coli* K12. The sequence of *valV* is shown and the differences to *valW* are indicated by arrows and the corresponding nucleotides of *valW*. An open circle indicates one nucleotide less in the D-loop.

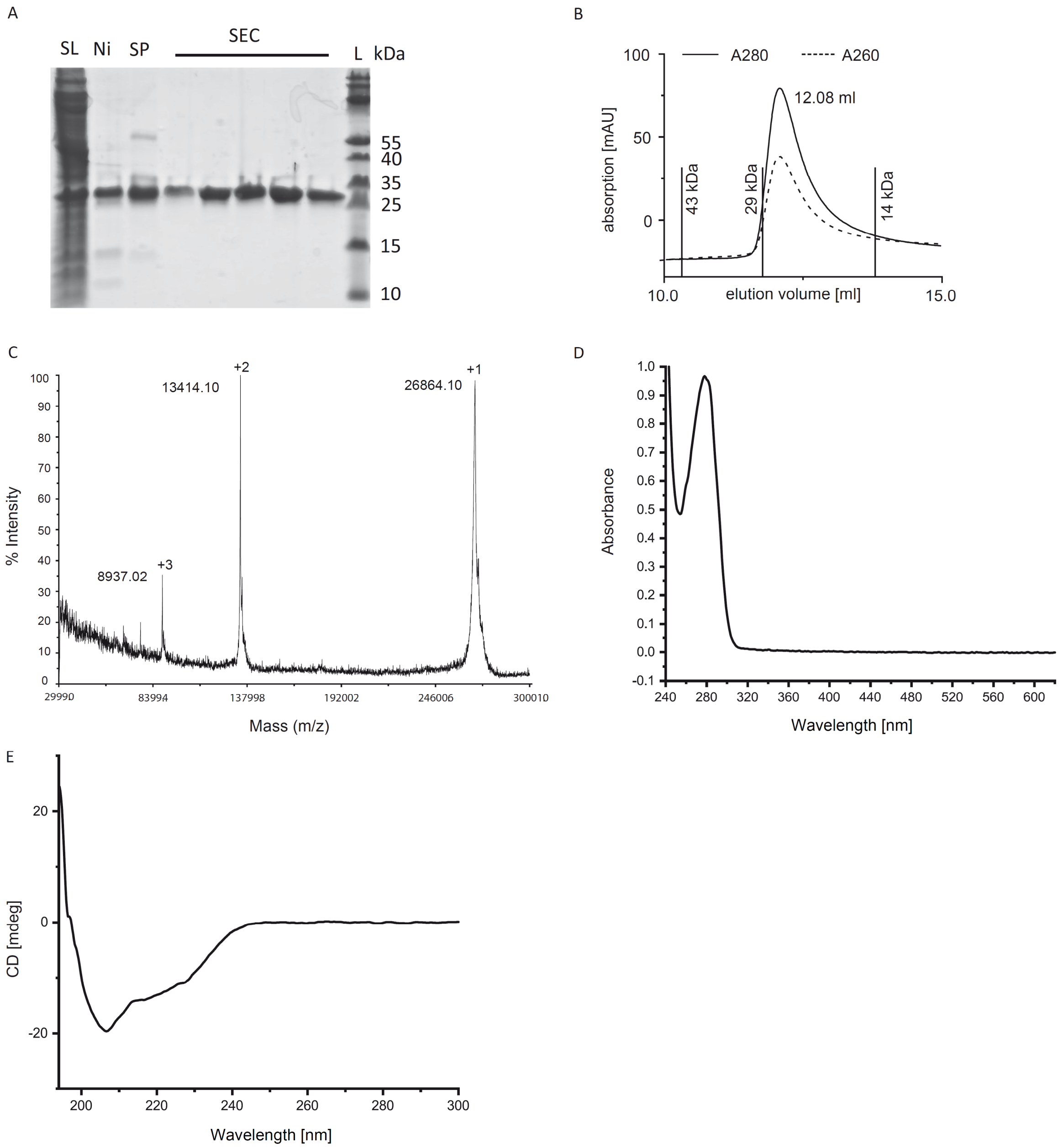

Supplementary Figure S2: Purification and characterization of *E. coli* YfiP **(A)** SDS-PAGE of the YfiP purification SL: supernatant lysate; Ni: pooled fractions after NiNTA affinity chromatography; SP: pooled fractions after cation exchange chromatography; SEC: YfiP-containing fractions from size exclusion chromatography; L: prestained protein ladder (Thermo Scientific™). **(B)** Analytical size exclusion chromatography (SEC) of *E. coli* YfiP, which elutes at ~12 ml on a Superdex 75 10/300 GL column (GE Healthcare). The absorption is shown at 280 and 260 nm. Elution volumes of molecular size standards are indicated. In comparison with the size standards, the elution volume of YfiP (expected mass monomer: 26.84 kDa) corresponds to a monomer with a molecular weight of ~26.24 kDa. **(C)** MALDI-TOF spectrum of purified full-length YfiP. The observed mass is in agreement with the expected mass of 26.84 kDa. **(D)** UV-Vis spectrum of 370 µM purified YfiP recorded at a Nanodrop2000c spectrophotometer (Thermo Fisher Scientific). The absorbance profile does not indicate the presence of a bound iron sulfur cluster or of bound SAM. **(E)** CD spectrum of 6.5 µM YfiP at 20°C. The spectrum was recorded in 50 mM sodium phosphate and 50 mM NaCl, pH 7.5 at a Jasco J-810 spectropolarimeter (Jasco) and clearly indicates a well-folded protein.

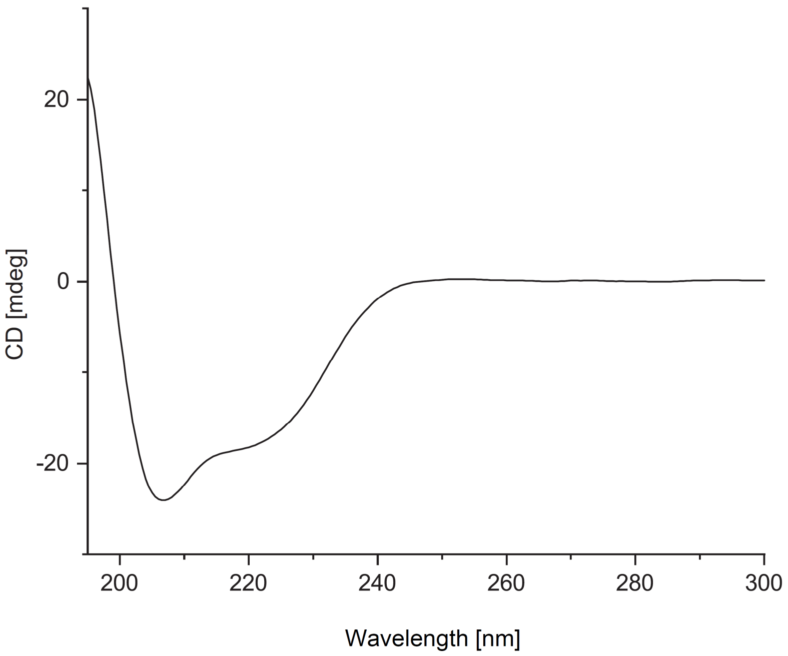

Supplementary Figure S3: Heterologous expression of the YfiP homolog from *C. saccharoperbutylacetonicum* in *E. coli* yields a well-folded protein. CD spectrum of 15 µM *C. saccharoperbutylacetonicum* YfiP at 20°C. The spectrum was recorded in 25 mM sodium phosphate and 25 mM NaCl, pH 7.5 with a Jasco J-810 spectropolarimeter (Jasco).

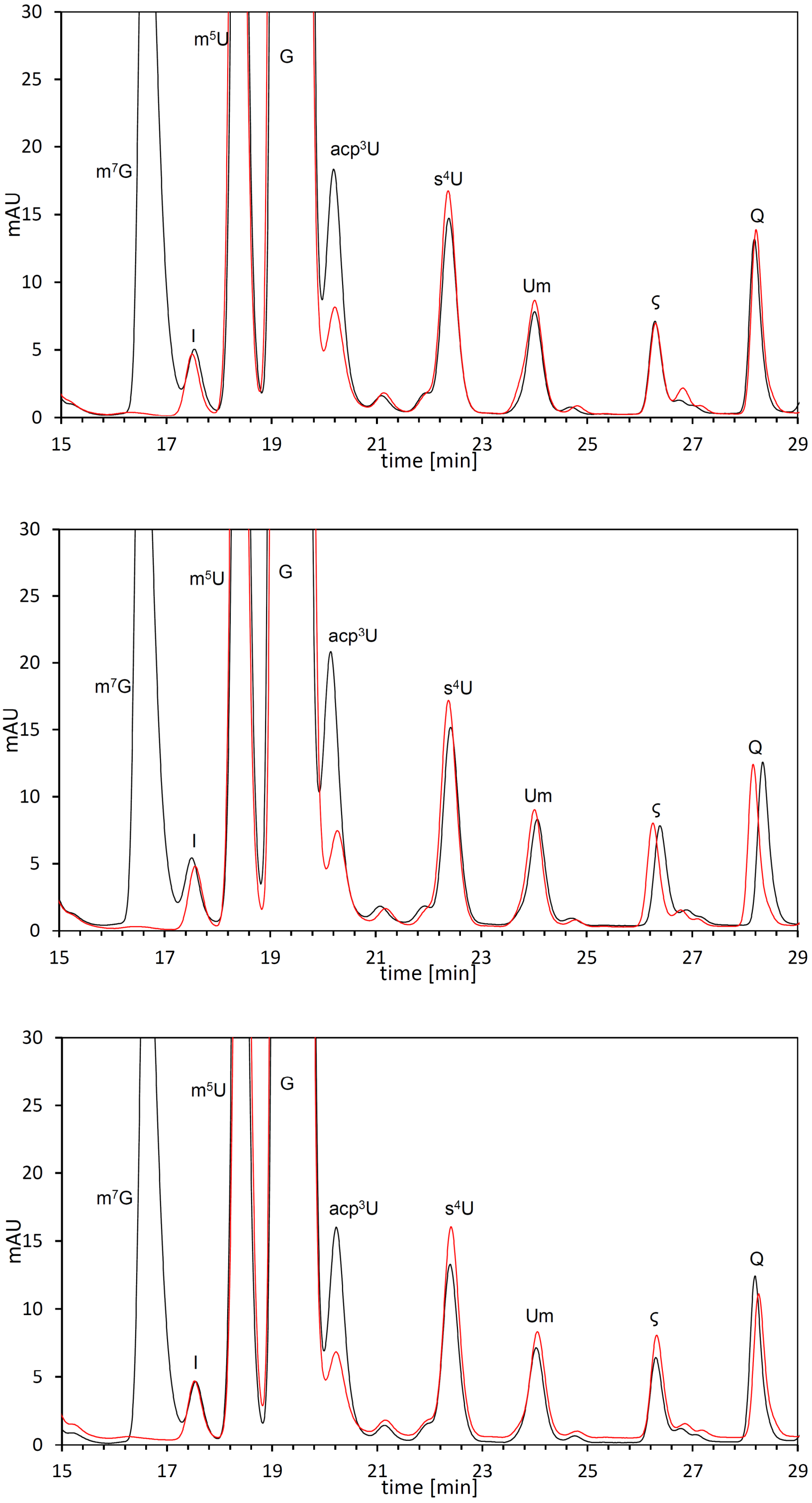

Supplementary Figure S4: Individual RP-HPLC elution profiles of tRNA nucleosides from wild type (black) and *ΔtrmB* deleted (red) *E. coli* cells corresponding to three additional biological replicates. Q, queuosine; ς , epoxyqueuosine.

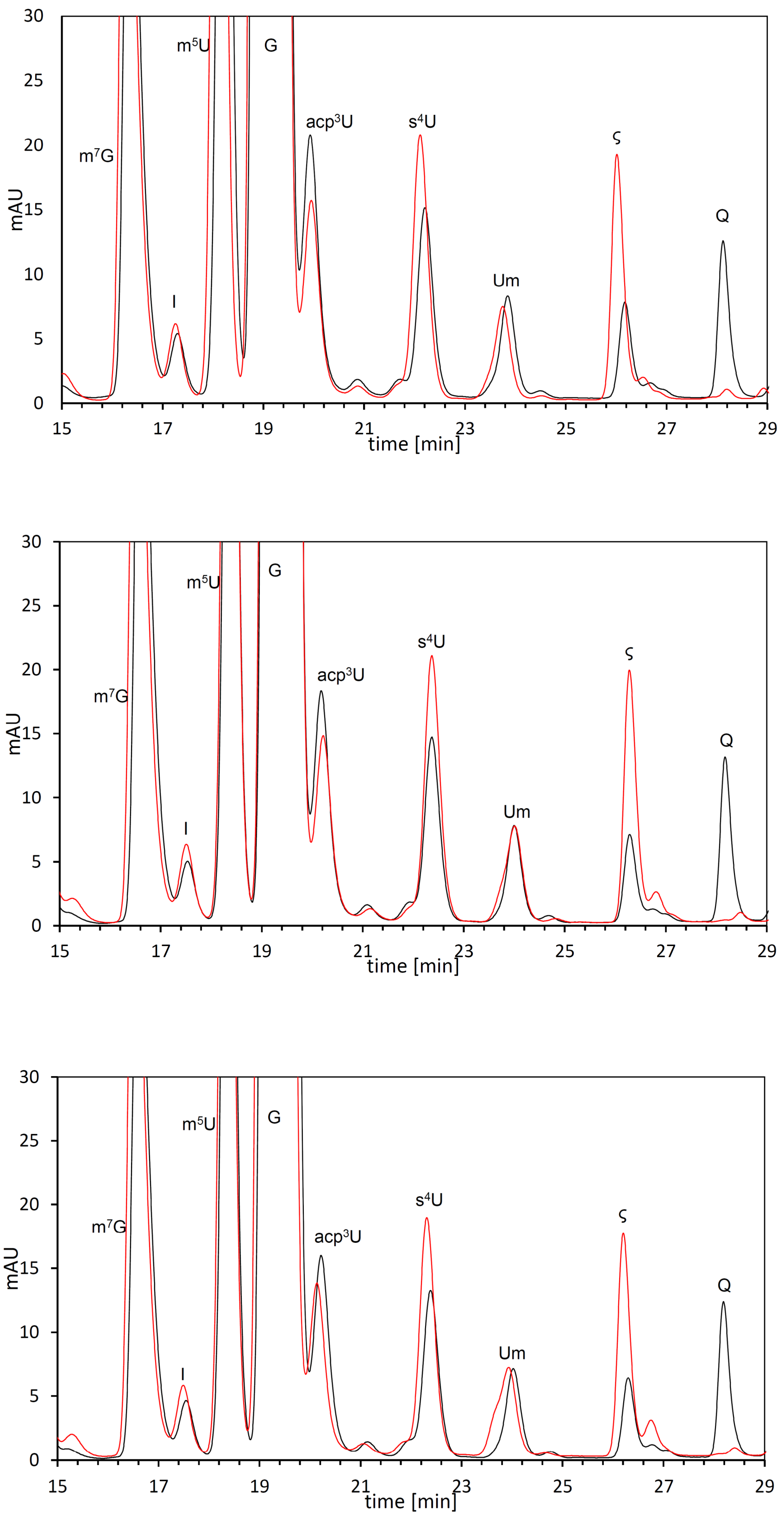

Supplementary Figure S5: Individual RP-HPLC elution profiles of tRNA nucleosides from wild type *E. coli* cells cultivated in LB (black) or M9 (red) medium corresponding to three additional biological replicates (n=3). Q, queuosine; ς , epoxyqueuosine.

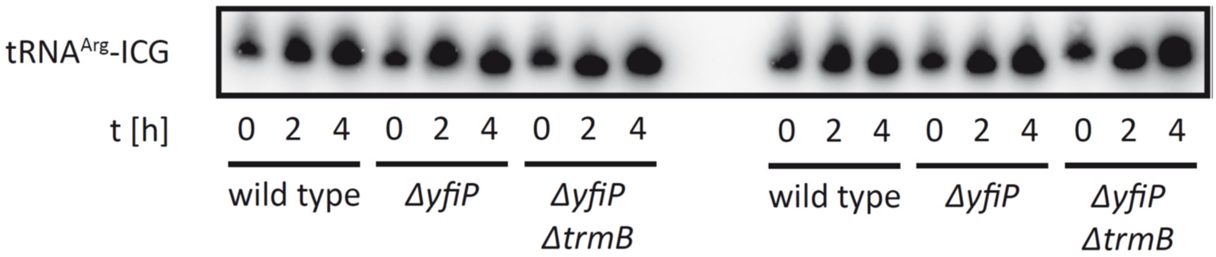

Supplementary Figure S6: The stability of the tRNA^{Arg}-ICG was analyzed by northern blotting as described for Figure 9B. Two additional biological replicates of each strain (wildtype, *ΔyfiP* and *ΔyfiP ΔtrmB*) were cultivated with the transcription inhibitors rifampicin and nalidixic acid for the indicated amounts of time (hours). See also figure caption to Figure 9B.

Supplementary Table S1: *E. coli* tRNAs described in this study. tRNA numbers: genomic tRNA database *E. coli* K12 (http://gtrnadb2009.ucsc.edu/Esch_coli_K12/)

**Isotype**	**tRNAdb**	**Anticodon**	**acp^{3}U (Modomics)**	**Oligo primer extension**
Val	#16	GAC	yes	tVal-8600
Val	#17	GAC	yes	tVal-8601
Val	#10,12,23,24,25	VAC	no	tVal-8609
Met	#82,86	MAU	yes	tMet-8666
Lys	#9,11,13,14,15,26	SUU	yes	tLys-8610
Ile	#40,57	GAU	yes	tIle-8641
Arg	#64,65,66,67	ICG	yes	tArg-8651
Phe	#30,55	GAA	yes	tPhe-8639

Supplementary Table S2: *E. coli* strains

**Strain**	**Genotype**[a]	**Source**
DH5α	*F- φ80dlacZΔM15 endA1 recA1 hsdR17(rk-,mk+) supE44 thi-1 gyrA96(Nalr) relA1 Δ(lacZYA-argF)U169 λ-*	Invitrogen
BL21(DE3)Gold	*F- ompT hsdS(rB-,mB-) dcm+ TetR gal λ(DE3) endA Hte E. coli B*	Agilent
BW25993	*λ$^-$ lacI^q Δ(araD-araB)567 rph-1 Δ(rhaD-rhaB)568 hsdR514 (CGSC #7693)*	(1), CGSC
Ec.PK5-11	*λ$^-$ lacI^q Δ(araD-araB)567 rph-1 Δ(rhaD-rhaB)568 hsdR514* ***ΔyfiP(4,678)::FRT-kan-FRT***	this study
Ec.PK6-10	*λ$^-$ lacI^q Δ(araD-araB)567 rph-1 Δ(rhaD-rhaB)568 hsdR514* ***ΔyfiP(4,678)::FRT***	this study
Ec.PK11-1	*λ$^-$ lacI^q Δ(araD-araB)567 rph-1 Δ(rhaD-rhaB)568 hsdR514* ***ΔyfiP(4,678)::FRT ΔtrmB(4,699)::FRT-kan-FRT***	this study
Ec.PK14-4	*λ$^-$ lacI^q Δ(araD-araB)567 rph-1 Δ(rhaD-rhaB)568 hsdR514* ***ΔyfiP(4,678)::FRT ΔtrmB(4,699)::FRT***	this study
Ec.PK12-1	*λ$^-$ lacI^q Δ(araD-araB)567 rph-1 Δ(rhaD-rhaB)568 hsdR514* ***ΔtrmB(4,699)::FRT-kan-FRT***	this study
Ec.PK15-1	*λ$^-$ lacI^q Δ(araD-araB)567 rph-1 Δ(rhaD-rhaB)568 hsdR514* ***ΔtrmB(4,699)::FRT***	this study

[a] Relevant genotypes are in bold. Numbers in parentheses behind *Δyfip* and *ΔtrmB* indicate base pairs removed from the corresponding wt gene (ATG = 1), CGSC (*E. coli* Genetic Stock center, cgsc2.biology.yale.edu)

Supplementary Table S3: Oligonucleotides

Name	Sequence 5' – 3' [a]	Description
yfiP-H1P4	*GCCCCGTTTTCTCCTCTGCGACAATGGCGTTTTTTCGACGCTCTCTTATG***ATTCCGGGGATCCGTCGACC**	PCR on pKD13
yfiP-H2P1	*CGGTCTCCCGCAGAAGTGACCGAATGATTTTAAACGCTTTCTAACTGTTC***TGTAGGCTGGAGCTGCTTCG**	PCR on pKD13
yfiP-A1	CTTGTTTGACGGAGAGGTG	verification
yfiP-A4	CAGACCTTGCCAGGGAGCC	verification
kan-A3	CAGTCATAGCCGAATAGCCTC	verification
kan-A2	GGATTCATCGACTGTGGCCG	verification
trmI-H1P4	*TGGATAATGCCCGTTTTCAGAACACTTTCACAAGCGACTAAACCTTTATG***ATTCCGGGGATCCGTCGACC**	PCR an pKD13
trmI-H2P1	*ACGCAGACGACGGCTACGGTTCTTTGCCATTATTTCACCCTCTCGAACAT***TGTAGGCTGGAGCTGCTTCG**	PCR an pKD13
trmI-A1	GCAACATCACTTCTGAGAGCC	verification
trmI-A4	AGCCACTTACGCACAATCG	verification
yfiP-GR4	CATCACCATCACCATCACGGATCTGAGAATCTTTATTTTCAGGGA**ACCGAAAACGCTGTTCTCCAG**	YfiP in pPK894 by gap-repair → pPK895
yfiP-GR5	ACTGGATCTATCAACAGGAGTCCAAGCTCAGCTAATTAAG**TTAAACGCTTTCTAACTGTTCTG**	
yfiP-11	GGTAATCGCGTTCGTCGCT**CA**CAACGCT**C**TCTTTTGCCAGAGAAATTATGTC	YfiP C31S C34S SPRINP mutagenesis → pPK935
yfiP-12	GACATAATTTCTCTGGCAAAAGA**G**AGCGTTG**TG**AGCGACGAACGCGATTACC	
yfiP-13	GTCTTTTGCCAGAGAAATTAT**C**TCTCT**C**TTCGACAATTACCCCAGCAC	YfiP C41S C43S SPRINP mutagenesis → pPK936
yfiP-14	GTGCTGGGGTAATTGTCGAA**G**AGAGA**G**ATAATTTCTCTGGCAAAAGAC	
yfiP-15	GTTTATCATGCTCGATGGTACC**GC**GCCGGAAGCTCGCAAGATG	YfiP W140A SPRINP mutagenesis →pPK937
yfiP-16	CATCTTGCGAGCTTCCGGC**GC**GGTACCATCGAGCATGATAAAC	
yfiP-17	CCGCTGTTTATCATGCTCG**C**TGGTACCTGGCCGGAAGC	YfiP D137A SPRINP mutagenesis →pPK1024
yfiP-18	GCTTCCGGCCAGGTACCA**G**CGAGCATGATAAACAGCGG	
tArg-GR1	ACGGTATCGATAAGCTTGATATCGAATTCCTGCAGCCCGGGCATCCGTAGCTCAGCTGGATAGAGTACTCGGCTACGA	$tRNA^{Arg}$ICG by gap repair in pRS423 → pPK963
tArg-GR2	ACCGAGCGGTCGGAGGTTCGAATCCTCCCGGATGCACCAAGTTCTAGAGCGGCCGCCACCGCGGTGGAGCTCCAGCTTT	
tArg-GR3	TTGGTGCATCCGGGAGGATTCGAACCTCCGACCGCTCGGTTCGTAGCCGAGTACTCTATCCAGCTGAGCTACGGATGCCC	
yfiP-GR16	CATCACCATCACCATCACGGATCTGAGAATCTTTATTTTCAGGGA**TTTCTTGCCCGCGGTAATCGC**	yfiP-GR5/yfiP-GR16, YfiP(21-232) in pPK894 by gap-repair → pPK938
yfiP-GR17	CATCACCATCACCATCACGGATCTGAGAATCTTTATTTTCAGGGA**TTGCTGATGTTCGACACCGAG**	yfiP-GR5/yfiP-GR17, YfiP(56-232) in pPK894 by gap-repair → pPK939
yfiP-GR18	CATCACCATCACCATCACGGATCTGAGAATCTTTATTTTCAGGGA**CTCATTGCTGATATTTTGCCTG**	yfiP-GR5/yfiP-GR18, YfiP(73-232) in pPK894 by gap-repair → pPK940

Vp-yfiP-GR5	CATCACCATCACCATCACGGATCTGAGAATCTTTATTTTCAGGGA**CCTCATGCCGTATCGCTGC**	Vp-YfiP in pPK894 by gap-repair → pPK955
Vp-yfiP-GR6	ACTGGATCTATCAACAGGAGTCCAAGCTCAGCTAATTAAG**TTAGGATGGCCGTAAGCCCGC**	
Cs-yfiP-GR2	AGCAGCCAACTCAGCTTCCTTTCGGGCTTTGTTAGCAGCCGGATC**TCAGTTAGACAGTTTAACGCCATTC**	Cs-YfiP in pPK565 by gap repair → pPK950
Cs-yfiP-GR4	TAGAAATAATTTTGTTTAACTTTAAGAAGGAGATATACAT**ATGGAATCAGAGTTCAAAGTG**	
Cs-yfiP-GR5	CATCACCATCACCATCACGGATCTGAGAATCTTTATTTTCAGGGA**GAATCAGAGTTCAAAGTGAAAC**	Cs-YfiP in pPK894 by gap-repair → pPK956
Cs-yfiP-GR6	ACTGGATCTATCAACAGGAGTCCAAGCTCAGCTAATTAAG**TCAGTTAGACAGTTTAACGCCATTC**	
yfiP-GR20	TAGAAATAATTTTGTTTAACTTTAAGAAGGAGATATACAT**ATGACCGAAAACGCTGTTCTC**	YfiP-6xHis in pPK565 by gap-repair → pPK984
yfiP-GR21	AGCAGCCAACTCAGCTTCCTTTCGGGCTTTGTTAGCAGCCGGATC**TTAGTGATGGTGATGGTGATGACTGCCAACGCTTTCTAACTGTTCTGC**	
tVal-8601	TGGTGCGTCCGAGTGGAC	primer extension $tRNA^{Val}$GAC
tVal-8600	TGGTGCGTTCAATTGGAC	primer extension $tRNA^{Val}$GAC
tVal-8609	TGGTGGGTGATGACGGGATC	primer extension $tRNA^{Val}$TAC
tMet-8666	TGGTGGCTACGACGGGATTC	primer extension $tRNA^{Met}$MAU
tMet-8615	TGGTGGCCCCTGCTGGAC	primer extension $tRNA^{Ile}$CAU
tIle-8641	TGGTAGGCCTGAGTGGAC	primer extension $tRNA^{Ile}$GAU
tLys-8610	TGGTGGGTCGTGCAGGATTC	primer extension $tRNA^{Lys}$SUU
tArg-8651	TGGTGCATCCGGGAGGATTC	primer extension $tRNA^{Arg}$ICG
tArg-8606	TGGTGTCCCCTGCAGGAATC	primer extension $tRNA^{Arg}${CU
tPhe-8639	TGGTGCCCGGACTCGGAATC	primer extension $tRNA^{Phe}$GAA
tArg-8651-Cy5	[Cy5]TGGTGCATCCGGGAGGATTC	primer extension $tRNA^{Arg}$ICG
tArg-8606-Cy5	[Cy5]TGGTGTCCCCTGCAGGAATC	primer extension $tRNA^{Arg}${CU
tRNA Arg ICG	[Btn]AAATGGTGCATCCGGGAGGATTCGAACCTCCGACCGCTCGGTTCGT	Isolation of tRNA isoacceptors
tRNA Lys yUU	[Btn]AAATGGTGGGTCGTGCAGGATTCGAACCTGCGACCAATTGATTAAA	Isolation of tRNA isoacceptors
tRNA Ile GAU	[Btn]AAACCTGAGTGGACTTGAACCACCGACCTCACCCTTATCAGGGGTGC	Isolation of tRNA isoacceptors
tRNA Met CAU	[Btn]AAATACGACGGGATTCGAACCTGTGACCCCATCATTATGAGTGATGT	Isolation of tRNA isoacceptors

[a] [CY5] cyanine5, [BTN] biotin labelled oligonucleotides

*oligonucleotides used for deletion of the *trmB* gene in *E. coli*

Supplementary Table S4: Plasmids

Plasmid	Description	Source
pKD46	*araC*_P_{araB}-promoter-λ Red genes (*exo*, *bet*, *gam*), Amp^R, $repA101^{ts}$	(1)
pKD13	FRT-kan^R-FRT, Amp^R, oriR6Kgamma	(1)
pCP20	*λ*rep^{ts}-*flp*, AmpR, cmR, pSC101(ori), $repA101^{ts}$	(1)
pRS423	*E. coli*/*S. cerevisiae* shuttle vector; ori, AmpR, 2μ-ori, *HIS3*	(2)
pRS425	*E. coli*/*S. cerevisiae* shuttle vector; ori, AmpR, 2μ-ori, *LEU2*	(2)
pQE9	Bacterial vector for expressing N-terminally 6xHis-tagged proteins, AmpR	Qiagen
pPK565	pRS425 (XhoI/SacI) + 1994bp PCR product. of pET11a (XhoI/SacI) $T7_{pro}$-$T7_{term}$-*lacI*, ori, AmpR, 2μ-ori, *LEU2*	this study
pPK894	pRS425(PvuII) + 1404bp *lacI* of pPK565 + 1232bp T5-lac-H6-TEV-rrnB-T1 of pQE9 PCR products by gap-repair	this study
pPK895	pPK894(BamHI/HindIII) + **6xHis-TEV-YfiP** PCR product by gap-repair	this study
pPK935	pPK895 after *in vitro* mutagenesis **YfiP(C31S C34S)**	this study
pPK936	pPK895 after *in vitro* mutagenesis **YfiP(C41S C43S)**	this study
pPK937	pPK895 after *in vitro* mutagenesis **YfiP(W140A)**	this study
pPK1024	pPK895 after *in vitro* mutagenesis YfiP(D137A)	this study
pPK938	pPK894(BamHI/HindIII) + **YfiP(aa21-232)** PCR product by gap-repair	this study
pPK939	pPK894(BamHI/HindIII) + **YfiP(aa56-232)** PCR product by gap-repair	this study
pPK940	pPK894(BamHI/HindIII) + **YfiP(aa73-232)** PCR product by gap-repair	this study
pPK950	pPK565(BamHI) + ***Cs*YfiP** PCR product by gap-repair	this study
pPK955	pPK894(BamHI/HindIII) + ***Vp*YfiP** PCR product by gap-repair	this study
pPK956	pPK894(BamHI/HindIII) + ***Cs*YfiP** PCR product by gap-repair	this study
pPK975	pPK894(StuI)+ argU-5'-**argV**-argU-3' PCR products by gap-repair	this study
pPK976	pPK895(StuI)+ argU-5'-**argV**-argU-3' PCR products by gap-repair	this study
pPK984	pPK565(BamHI) + **YfiP-6xHis** PCR product by gap-repair	this study
pPK963	pRS423(BamHI) + **tArg(ICG)** 3 oligos by gap-repair	this study

Supplementary Table S5: Zinc content of Yfip homologs by ICP-MS

Protein	Concentration [μM]		Molar ratio Zinc:Protein
	Protein	Zinc	
E. coli YfiP	450	429	0.95
Clostridium YfiP	8.4	7.1	0.85
Pyrococcus Nob1	167	140	0.84
Legionella Lpp1663	370	0.377	0.001

Supplementary Table S6: Quantification of the modified nucleosides acp^{3}U and m^{7}G from three independent biological replicates by measuring the ratios between the integrated peak areas from the respective HPLC chromatograms shown in Fig. S4 and Fig. S5 for the acp^{3}U signal and the total peak area over all peaks in the chromatogram (acp^{3}U/total) or between the acp^{3}U signal and the individual signals for the unmodified U, C, G and A nucleosides, respectively. For comparison, the relative amount of m^{7}G in each chromatogram was also quantified in this way. No m^{7}G is present in the *ΔtrmB* samples.

	**WT (LB) [10^{-2} area]**	***ΔtrmB* (LB) [10^{-2} area]**	**WT (M9) [10^{-2} area]**	***ΔtrmB*/WT (LB) [%]**	**WT (M9/LB) [%]**
**acp^{3}U/total**	0.232±0.022	0.098±0.008	0.182±0.007	42.2	78.6
**acp^{3}U/U**	1.493±0.142	0.623±0.052	1.202±0.041	41.8	80.5
**acp^{3}U/C**	1.321±0.139	0.556±0.050	1.040±0.047	42.1	78.8
**acp^{3}U/G**	0.657±0.064	0.273±0.024	0.517±0.020	41.6	78.8
**acp^{3}U/A**	0.913±0.082	0.385±0.035	0.719±0.030	42.1	78.8
**m^{7}G/total**	0.669±0.035		0.709±0.018		106.0
**m^{7}G/U**	4.312±0.231		4.679±0.088		108.5
**m^{7}G/C**	3.814±0.236		4.050±0.134		106.2
**m^{7}G/G**	1.897±0.107		2.013±0.050		106.1
**m^{7}G/A**	2.637±0.126		2.801±0.081		106.2

Supplementary Table S7: Parameters of DMRM (dynamic multiple reaction monitoring) method for detection and quantification of modified nucleosides.

	compound	Precursor Ion	Product Ion	Ret Time (min)	Delta Ret Time	Fragmentor	Collision Energy	Cell Accelerator Voltage	Polarity
unla [illegible]	A	268.1	136.1	5.1	1	120	40	5	Positive
	acp^{3}U	346.1	214.1	2.2	1.5	90	10	5	Positive

	C	244.1	112.1	2	1	120	40	5	Positive
	D	247.1	115.1	1.7	1	90	5	5	Positive
	G	284.1	152.1	4	1	120	40	5	Positive
	Gm	298.1	152.1	4.8	1	100	10	5	Positive
	I	269.1	137.1	3.8	1	100	10	5	Positive
	m^1G	298.1	166.1	4.7	1	105	13	5	Positive
	m^2A	282.1	150.1	6.2	1	120	20	5	Positive
	m^7G	298.1	166.1	3.5	1.5	105	14	5	Positive
	U	245.1	113.1	3	1	100	15	5	Positive
bact SILIS	A	283.1	146.1	5.1	1	120	40	5	Positive
	acp^3U	362.2	225.1	2.2	1.5	90	10	5	Positive
	C	256.1	119.1	2	1	120	40	5	Positive
	D	258.1	121.1	1.7	1	90	5	5	Positive
	G	299.1	162.1	4	1	120	40	5	Positive
	Gm	314.1	162.1	4.8	1	100	10	5	Positive
	I	283.1	146.1	3.8	1	100	10	5	Positive
	m^1G	314.1	177.1	4.7	1	105	13	5	Positive
	m^2A	298.1	161.1	6.2	1	120	20	5	Positive
	m^7G	314.1	177.1	3.5	1	105	14	5	Positive
	U	256.1	119.1	3	1	100	15	5	Positive

Supplementary References

1. Datsenko, K.A. and Wanner, B.L. (2000) One-step inactivation of chromosomal genes in *Escherichia coli* K-12 using PCR products. *Proc. Natl. Acad. Sci. USA,* **97,** 6640–6645.
2. Christianson, T.W., Sikorski, R.S., Dante, M., Shero, J.H. and Hieter, P. (1992) Multifunctional yeast high-copy-number shuttle vectors. *Gene,* **110,** 119–122.
